# Barnacle: detecting and characterizing tandem duplications and fusions in transcriptome assemblies

**DOI:** 10.1186/1471-2164-14-550

**Published:** 2013-08-14

**Authors:** Lucas Swanson, Gordon Robertson, Karen L Mungall, Yaron S Butterfield, Readman Chiu, Richard D Corbett, T Roderick Docking, Donna Hogge, Shaun D Jackman, Richard A Moore, Andrew J Mungall, Ka Ming Nip, Jeremy DK Parker, Jenny Qing Qian, Anthony Raymond, Sandy Sung, Angela Tam, Nina Thiessen, Richard Varhol, Sherry Wang, Deniz Yorukoglu, YongJun Zhao, Pamela A Hoodless, S Cenk Sahinalp, Aly Karsan, Inanc Birol

**Affiliations:** 1 Canada’s Michael Smith Genome Sciences Centre, British Columbia Cancer Agency, Vancouver, Canada; 2 School of Computing Science, Simon Fraser University, Burnaby, Canada; 3 Terry Fox Laboratory, British Columbia Cancer Agency, Vancouver, Canada; 4 Department of Medical Genetics, University of British Columbia, Vancouver, Canada; 5 Computer Science and Artificial Intelligence Laboratory, Massachusetts Institute of Technology, Cambridge, USA

**Keywords:** Transcriptome assembly, Chimeric transcripts, Fusion, Partial tandem duplication, PTD, Internal tandem duplication, ITD, RNA-seq, Transcriptome

## Abstract

**Background:**

Chimeric transcripts, including partial and internal tandem duplications (PTDs, ITDs) and gene fusions, are important in the detection, prognosis, and treatment of human cancers.

**Results:**

We describe Barnacle, a production-grade analysis tool that detects such chimeras in de novo assemblies of RNA-seq data, and supports prioritizing them for review and validation by reporting the relative coverage of co-occurring chimeric and wild-type transcripts. We demonstrate applications in large-scale disease studies, by identifying PTDs in MLL, ITDs in FLT3, and reciprocal fusions between PML and RARA, in two deeply sequenced acute myeloid leukemia (AML) RNA-seq datasets.

**Conclusions:**

Our analyses of real and simulated data sets show that, with appropriate filter settings, Barnacle makes highly specific predictions for three types of chimeric transcripts that are important in a range of cancers: PTDs, ITDs, and fusions. High specificity makes manual review and validation efficient, which is necessary in large-scale disease studies. Characterizing an extended range of chimera types will help generate insights into progression, treatment, and outcomes for complex diseases.

## Background

**Figure 1:**
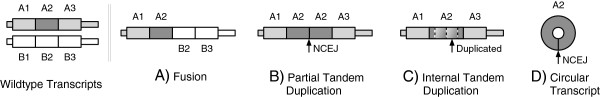
**Chimeric transcript event types. A)** A fusion in which the first two exons of gene A are joined to the last two exons of gene B. **B)** A partial tandem duplication in which the second exon of gene A is duplicated. NCEJ marks the non-canonical exon junction between the two copies of exon A2. **C)** An internal tandem duplication in which a portion of the second exon of gene A is duplicated, internal to the exon. **D)** A circular transcript involving only the second exon of gene A. Note that it contains the same A2-A2 NCEJ as the PTD in **(B)**.

A chimeric transcript is an RNA molecule that does not have a collinear mapping to a single reference gene model. Such a transcript can result from genome rearrangement events that occur at the DNA level, or transcriptome events that occur at the RNA level [1]. Two types of chimeric transcripts that are important in human cancers are fusions (e.g. [2]), in which parts of two genes located on the same or on different chromosomes are joined (Figure 1A); and tandem duplications, in which part of a gene is repeated. A tandem duplication can be further classified as either a partial tandem duplication (PTD, e.g. [3]), if both edges of the duplicated segment correspond to annotated exon boundaries that are involved in splicing (Figure 1B); or an internal tandem duplication (ITD, e.g. [4]) otherwise (Figure 1C). The defining characteristic of a PTD is a non-canonical exon junction (NCEJ): a junction from the end of an exon A, to the beginning of the same exon or of another exon that is 5 prime of exon A in the reference isoform(s) (Figure 1B). Salzman et al. [5] present evidence for circular transcripts (Figure 1D), which can produce NCEJs that are identical to those seen in PTDs; as discussed in that work, RNA-seq data cannot support differentiating between linear PTD transcripts and circular transcripts when the total length of the exons involved is greater than the fragment-length range of the sequencing experiment. However, a poly(A)-selected RNA-seq library preparation protocol should enrich for linear transcript products. Al-Balool et al. [6] performed a large-scale experimental analysis of post-transcriptional exon shuffling (PTES) events detected as NCEJs in human datasets, along with extensive wet-lab validations; some of the NCEJs that they reported could represent PTDs.

Chimeric transcripts have been detected in a range of eukaryotes [7-10], including mice [11], rats [12,13], and both healthy and diseased human tissues [14-16]. Although Al-Balool et al. [6] reported four genes in which expression levels of NCEJs were greater than 50% of those of their corresponding wild-type transcripts, the majority of the 72 expressed NCEJ sequences that they validated were expressed at low relative levels. The functional importance of chimeric transcripts in healthy tissues remains controversial; such transcripts generally have low relative expression levels, and no high-throughput tools have been available for estimating the expression levels of such transcripts [1,6].

Specific tandem duplications and fusions are important in detecting, prognostically scoring, and treating cancers (e.g. in AML, MLL PTDs [3]; FLT3 ITDs [4]; PML/RARA fusions [2]). In cancerous tissues, chimeras are often the result of genomic events; however, Kannan et al. [14] found strong evidence for transcriptome-level production of chimeric transcripts in prostate cancer samples. They detected many more events in cancer samples than in matched benign samples, and a large fraction of their detected events were either specific to, or had a much higher expression level in, the cancer samples. Similarly, Li et al. [17] found evidence in normal endometrial tissue of a fusion transcript due to regulated trans-splicing that is identical to a constitutively expressed fusion transcript resulting from a chromosomal translocation in endometrial tumours. Schnittger et al. [18] found that, beyond the simple presence or absence of an ITD in the FLT3 gene, the relative expression of chimeric FLT3 transcripts relative to wild-type FLT3 can be used as a prognostic indicator.

Transcriptome sequencing (RNA-seq) can support high-throughput detection of chimeric transcripts. De novo transcriptome assembly of RNA-seq data (e.g. [19-21]) generates contigs representing transcripts without relying on annotated transcript models or assuming collinearity between transcripts and the genome, and so is well suited to discovering novel transcript structures. Further, the longer sequences assembled from reads yield alignments that have specific signatures, facilitating detection and characterization of contigs that cannot be explained by reference gene models, and that may reflect complex genomic or transcription-related events.

Tools like AGE [22] and DELLY [23] predict a range of structural variations, including tandem duplications, in genomic data. A number of tools are available for detecting fusions in RNA-seq data (e.g. [24-27]), but do not predict tandem duplications. Yorukoglu et al. [28] developed Dissect, a novel tool for non-collinear alignments of long transcriptome sequences to a reference genome; however, this tool performs alignment only, and does not further characterize the alignments. To our knowledge, no production-grade, high-throughput tool is available that uses RNA-seq data to detect and characterize PTDs or ITDs, and compares the coverage of chimeric transcripts to their corresponding wild-type transcripts.

Here, we describe a discovery and analysis pipeline for Browsing Assembled RNA for Chimeras with Localized Evidence (Barnacle). It integrates evidence from a range of data types: (i) assembled transcriptome contigs and their alignments to a reference genome, (ii) read alignments to assembled transcriptome contigs, and (iii) gene and repeat annotations in the reference genome. By comparing assembled contigs to a reference genome sequence, Barnacle identifies a wide range of non-collinear alignment topologies, and currently characterizes three types of chimeric transcripts: NCEJs (which can represent PTDs, circular transcripts, or exon-shuffling events), ITDs, and fusions. Barnacle provides a range of filtering options, allowing users to adjust sensitivity and specificity to suit their applications. When provided with alignments of RNA-seq reads to a reference genome, Barnacle supports prioritizing predictions by comparing the coverages of candidate chimeric transcripts and the corresponding wild-type transcripts.

We applied this tool to two deeply sequenced acute myeloid leukemia (AML) samples. Among the events that we predicted are several known to be important in AML: a PTD in the myeloid/lymphoid or mixed-lineage leukemia (MLL) gene [3]; two distinct ITDs in the fms-related tyrosine kinase 3 (FLT3) gene [4]; and a pair of reciprocal fusions between the promyelocytic leukemia (PML) and alpha retinoic acid receptor (RARA) genes [2]. These results for a well-studied cancer with known and clinically significant chimeric transcripts suggest that Barnacle will be a useful tool for detecting and characterizing such events in RNA-seq data, particularly in large-scale studies of complex diseases.

## Results and discussion

### Barnacle overview

**Figure 2:**
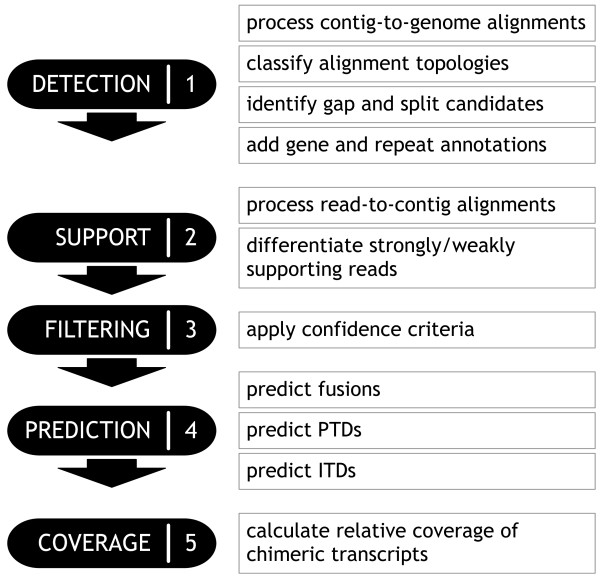
Stages of the Barnacle pipeline.

The Barnacle pipeline is composed of five stages (Figure 2). The first stage examines contig alignments to genomic sequences (contig-to-genome alignments) and identifies anomalous or non-reference (candidate) contigs that have a variety of alignment topologies. The second stage examines transcriptome read alignments to the assembled contig sequences (read-to-contig alignments), and calculates read support for these candidate contigs. The third stage applies user-specified filters to the candidate contigs and retains sufficiently confident candidates. The fourth stage identifies chimeric transcripts of particular types from the filtered candidates. These four stages require the following as input: contig sequences in FASTA format; contig-to-genome alignments in PSL format; read-to-contig alignments in BAM format; and gene and repeat annotations in UCSC genePredExt and BED file formats, respectively. The optional final stage uses read alignments to genomic sequences (read-to-genome alignments) in BAM format to compare the coverage of the predicted chimeric transcripts to their corresponding wild-type transcripts. Although we used the Trans-ABySS pipeline for the assemblies and alignments in our experiments below, Barnacle can be applied to the outputs of a variety of combinations of assembly and alignment tools, provided that the outputs can be converted to the accepted formats.

### Stage 1 Detecting candidate contigs

#### Stage 1.1. Candidate identification

**Figure 3:**
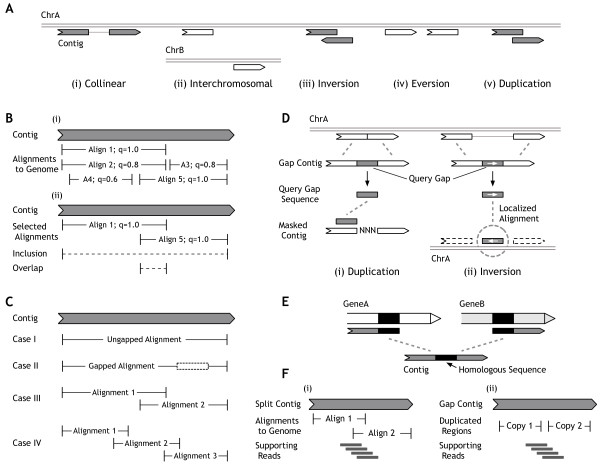
**Details of the Barnacle pipeline. A)** Contrasting a collinear alignment topology (i) with non-collinear topologies: (ii) interchromosomal, which involves alignment to two chromosomes; (iii) inversion, which involves alignment to two strands; (iv) eversion, which involves alignment with a reversal of block ordering; and (v) duplication, which involves multiple alignment to the same region. **B)** (i) Pieces of the contig can be aligned to different regions in the genome, with ‘q’ denoting the quality of each alignment, normalized to the range [0,1]. (ii) Alignments 1 and 5 are selected, because of their high qualities and inclusion, and their low overlap. **C)** Alignment selection can result in one of four cases: (i) a single ungapped alignment is selected, (ii) a single gapped alignment is selected, (iii) a pair of alignments is selected, or (v) more than two alignments are selected. **D)** In gap contigs a piece of the contig does not take part in the initial contig-to-genome alignment. Gap contigs are checked for duplications (i) by realigning the gap sequence back to the contig with the original gap location masked, and for inversions (ii) by realigning the gap sequence to a region of the genome determined by the original contig-to-genome alignment. **E)** Fusions can have homologous sequence near the breakpoint that makes it impossible to determine the precise breakpoint position. **F)** For split candidates (i), read support is calculated in the region surrounding the overlap of the two contig-to-genome alignments. For gap candidates involving a duplication (ii), read support is calculated in the region between the two copies of the duplicated sequence.

Barnacle begins by examining alignments of contigs to the reference genome, determining which alignment(s) best represent each contig, and comparing these alignments to the alignment signatures that we have identified as indicating non-collinear alignment topologies, such as interchromosomal, where parts of the contig align to different chromosomes; inversion, where parts of the contig align to different strands of the same chromosome; eversion, where parts of the contig align out of order to the same strand of the same chromosome; and duplication, where parts of the contig align to the same region of the same strand of the same chromosome (Figure 3A). While examining alignments, Barnacle uses their genomic coordinates to assign genes to each. Because long-sequence aligners (e.g. BLAT [29]) are designed to generate collinear alignments, determining which alignment result(s) most likely represent the true genomic source(s) of the transcript that produced a given contig is non-trivial. Barnacle cannot simply pick the alignment(s) with the highest alignment score or percent identity.

The alignment selection problem is as follows. By contig and genome we are referring to strings, S, of characters in the alphabet {a, c, g, t}. For any such string, we will refer to the substring of S starting at position i and ending at position j, inclusive, as S[i,j]. For a contig C, define a(C), a contig-to-genome alignment of C, as a mapping from each position i in C to one of:

•an insertion, showing that the base at contig position i is not aligned to the base in any genomic position;

•a match(j), showing that the base at contig position i is aligned with and the same as the base at genomic position j;

•a mismatch(j), showing that the base at contig position i is aligned with and different from the base at genomic position j.

Note that since we are considering RNA-to-DNA alignments from an RNA contig-centric perspective (i.e. we define an alignment as a single mapping for each position in a contig sequence), we are not concerned with deletions, i.e. unaligned genomic positions flanked by aligned genomic positions, which would typically represent introns in RNA-to-DNA alignments. As no contig position would be mapped to such deletions, we do not consider them.

We define a query gap as a set of adjacent contig positions mapped to an insertion (Figure 3D). That is, if for positions i and j, i < j, positions i-1 and j + 1 are marked as a match or a mismatch, and all positions between i and j, inclusive, are marked as an insertion, then C[i,j] is a query gap. Query gaps can also occur at either edge of a contig, when either i = 0 or j = |C|-1.

Now, for each contig C, we have a set of contig-to-genome alignments: A(C) (Figure 3B-i). We define the following functions for analysing sets of contig-to-genome alignments: quality(A(C)) is the sum of the qualities of the alignments in A(C), based on the alignment score and percent identity reported by the contig aligner; inclusion(A(C)) is the fraction of the positions in C marked as a match or mismatch in any of the alignments in A(C); overlap(A(C)) is the fraction of positions in C marked as match or mismatch in more than one of the alignments in A(C); and size(A(C)) is the number of alignments in A(C) (Figure 3B-ii). We then define score(A(C)) = quality(A(C)) + inclusion(A(C)) - overlap(A(C)) - size(A(C)). Our goal is to find all subsets A*(C) of A(C) that approximate a maximization of score(A*(C)).

There are four possible cases to consider for each alignment set A*(C) found for contig C (Figure 3C):

Case I: A*(C) is a single alignment that contains no query gaps; the contig is considered non-chimeric, and not processed further.

Case II: A*(C) is a single alignment that contains one or more query gaps; the contig is considered a potential gap-candidate (see below).

Case III: A*(C) is a pair of alignments; the contig is considered a potential split-candidate (see below).

Case IV: A*(C) contains more than two alignments; the contig is considered outside the current scope of Barnacle characterization, and not processed further.

Barnacle approaches the alignment selection problem heuristically. First, for a contig C, if any single alignment result a(C) in the set of alignments A(C), has inclusion(a(C)) greater than a user-specified threshold, then contig C is considered to fall into Case I and is not processed further. Otherwise, the alignments in A(C) are grouped by their pairwise overlap values, resulting in a partitioning of the positions in C based on the edges of the alignments in each group. That is, if A(C) = {a(C), b(C), c(C), …} and overlap({a(C),b(C)}) is greater than a user-specified threshold, then a(C) and b(C) are grouped together. Furthermore, if either overlap({a(C),c(C)}) or overlap({b(C),c(C)}) is greater than the threshold, c(C) is also grouped together with a(C) and b(C).

Barnacle then chooses the highest quality alignment result(s) in each alignment group. If there are multiple alignments in a single group that have qualities within a user-specified range of the highest quality alignment, then they are all marked as multi-mapping and considered in following steps. Candidates involving alignments marked as multi-mapping can be removed at the filtering stage, if desired (see below). By default, Barnacle discards any alignments to mitochondrial DNA, but the user can disable this option.

To determine whether a potential gap-candidate (involving a contig falling into Case II, see above) represents a chimeric transcript, Barnacle processes it as follows. First, the contig-to-genome alignment a(C) must pass user-specified thresholds for quality({a(C)}), inclusion({a(C)}), and the length of the query gap. If the alignment passes these thresholds, then for each query gap in that alignment, Barnacle attempts one or two local realignments, using the query gap sequence as the new query. The first realignment looks for duplications by attempting to align the query gap sequence to the sequence produced by masking the gap position in the original contig sequence (Figure 3D-i). If C[i,j] is the query gap, then Barnacle attempts an alignment between C[i,j] and C[0,i-1] + C[j + 1,|C|-1] (i.e. the concatenation of the sequence before and after the query gap). If the query gap is internal to the contig, i.e. i > 0 and j < |C|-1, then a second realignment is attempted. This alignment looks for inversions by attempting to align the query gap sequence to the genomic region bounded by the genomic alignment coordinates of the bases flanking the gap in the original contig-to-genome alignment (Figure 3D-ii). If the contig position C[i-1] is aligned to genomic position G[i’] and C[j + 1] is aligned to genomic position G[j’], then C[i,j] is aligned to G[min(i’,j’), max(i’,j’)]. If either of these realignments is successful, Barnacle creates a gap-candidate from the contig C, the original contig-to-genome alignment a(C), and the successful query-gap realignment a’(C[i,j]).

Potential split-candidates (involving Case III contigs, see above) need only have inclusion(A*(C)) greater than a user-specified minimum value for Barnacle to create a split-candidate from the contig C, and the pair of contig-to-genome alignments A*(C).

At this point, Barnacle has identified gap- and split-candidates with a variety of alignment topologies. Each candidate is made up of a contig and a pair of alignments associated with that contig. For gap-candidates these are a single contig-to-genome alignment and a gap-realignment; for split-candidates they are two contig-to-genome alignments. If Barnacle has marked either of these alignments as multi-mapping, the candidate contig is also marked as multi-mapping. A single contig can be present in multiple candidates if, for example, that contig multi-maps. Barnacle labels each candidate with the appropriate alignment topology and uses these labels while calculating support and annotations, and while identifying specific types of chimeric transcripts.

#### Stage 1.2. Candidate grouping

Because some transcriptome assemblers produce a meta-assembly, i.e. a set of contigs that has been produced by merging several independent assemblies generated with a range of assembly parameters [20,21], a single event can be represented by multiple contigs. Alternative splicing can also result in multiple contigs representing the same event. To address this, gap- and split-candidates are grouped by their genomic locations and alignment orientations.

#### Stage 1.3. Candidate annotation

For use in filtering, predictions, and characterization, several types of annotations are associated with each candidate, based on files provided by the user. If the genomic coordinates of a predicted event overlap any repeat regions, segmental duplications, or small structural RNA regions (such as tRNAs or snRNAs), the event is annotated with this information. Also, for each candidate Barnacle determines whether both, one, or neither of its genomic breakpoint coordinates match annotated exon boundaries. For a fusion from gene A to gene B, the first breakpoint is the last genomic position from gene A that is present in the transcript, while the second breakpoint is the first genomic position from gene B that is present in the transcript. Note that Barnacle is able to detect fusions that involve non-canonical exon junctions; it does not require that fusion breakpoints match annotated exon boundaries. For a tandem duplication event, the breakpoints are the first and last genomic positions of the duplicated segment.

### Stage 2 Calculating read support

When the two genomic regions connected by a chimeric breakpoint have high sequence homology, it can be 'impossible to unambiguously determine the exact position of that breakpoint within the homologous region (Figure 3E). Given this, a search region, P, surrounding the breakpoint is determined on the contig, based on the alignments associated with the candidate (Figure 3F-i). For gap candidates involving a duplication event, P is defined as the region between the two copies of the duplicated sequence (Figure 3F-ii). At each position p within P, Barnacle calculates the read depth R(p), counting only reads mapping to the contig without mismatches and overlapping p by at least a user-specified minimum on each side (e.g. 5 nucleotides (nt)). Barnacle reports the minimum value of R(p) over all positions p in the search region P as the read-to-contig support for the current candidate contig. This guarantees that wherever the breakpoint is within P, at least the reported number of read alignments overlap the breakpoint.

Since multiple contigs may represent the same event, Barnacle uses the following method for handling reads that map to multiple contigs. For a given read r, let C(r) represent the set of contigs that r maps to. For a contig C, let E(C) represent the set of contigs representing the same event as C, i.e. C plus any other contigs grouped together with C (see Stage 1.2 above). Now define score(r,C) = |intersection(C(r), E(C))| / |C(r)|. For all C in C(r), r strongly supports C if score(r,C) ≥  0.5, otherwise r weakly supports C. Barnacle reports both total read-to-contig support (i.e. including strongly supporting as well as any weakly supporting read alignments) and strong read-to-contig support (i.e. including only strongly supporting read alignments).

### Stage 3 Filtering candidates

Barnacle provides several filters that a user can apply to the candidates at this point. The filters and their default values were iteratively developed in close interaction with manual review. The following filtering criteria can be applied to each candidate, with default values shown in parentheses:

1. the number of candidate groups containing the contig associated with the candidate (fail contigs involved in more than 3 groups);

2. whether the candidate has been marked as multi-mapping (fail multi-mapping candidates);

3. whether the inferred event is a homopolymer sequence (fail homopolymer events);

4. whether the breakpoints occur within any repetitive regions (allow breakpoints in repeats);

5. whether the breakpoints occur within any structural RNA regions (fail breakpoints in structural RNAs);

6. the percent identities of the pair of alignments associated with the candidate (fail contigs with less than 99.0% identity for either alignment);

7. the total fraction of the bases in the contig marked as “match” or “mismatch” in the pair of alignments associated with the candidate (fail contigs with less than 0.9 of their positions marked as “match” or “mismatch”);

8. the amount of support from read-to-contig alignments (fail contigs with fewer than 5 strongly supporting reads);

9. the maximum amount of overlap in the contig coordinates of the pair of alignments associated with the candidate (fail contigs with more than 75 nt of overlap);

10.  whether the event might be a misalignment of the poly(A) tail of a transcript (fail poly(A) events).

The filters for the number of distinct candidate groups (1), contig multi-mapping (2), homopolymer sequences (3), annotated repeats (4), and small structural RNAs (5) all address the challenges that repetitive sequences pose to assembling and aligning contigs, which reduce prediction confidence. There are two situations that can cause a contig to create candidates in multiple candidate groups. First, the contig could represent a combination of multiple simultaneous events, such as a pair of fusions joining three genes into a single transcript. Second, alignment ambiguity can result in several mutually exclusive events that are explainable by the same contig. For example, if two genes A and A’ have similar sequences and there is a fusion between one of them and a third gene, B, then the assembled contig will have one piece that aligns to gene B, while the remainder of the contig aligns with similar qualities to both gene A and gene A’. So, while only one fusion actually occurred (either A/B or A’/B), the resulting contig will be explainable by either of them. Because the frequency of the events being detected is typically low compared to the transcriptome size, and contigs that involve multiple simultaneous events are more difficult to characterize, we allow the user to limit the number of events that a reported contig can contain. This filter (1) can also be used to roughly control the amount of contig multi-mapping allowed in the final predictions, since filter (2) removes contigs with any amount of multi-mapping. For a candidate to fail the homopolymer filter, it must be a gap-candidate, and every position within the realigned portion of the query gap must be the same base. Because expansions of homopolymer and repetitive regions can have signatures similar to general duplication events, we allow the user to filter out such expansions with filters (3) and (4) if desired. Small structural RNAs (such as tRNAs and snRNAs) can resemble repeats [30] and can be eliminated using filter (5).

Filters for percent identity (6) and contig inclusion (7, also see Stage 1.1) are used to filter candidates based on the confidence with which Barnacle chose alignments to represent the genomic source(s) of the contig associated with the candidate, when creating the candidate in Stage 1.

The read-support filter (8) specifies minimum values for total and strong read support calculated for each candidate in Stage 2. It can help avoid false positives due to misassembly, by requiring that reads directly support the novel sequence at the event breakpoint, and allows the user to adjust the sensitivity to weakly expressed chimeras by filtering predictions based on chimera read coverage. See Stage 2 above for a description of how read support is calculated. The discussion of the AML datasets below suggests how to select appropriate threshold values for this filter. This filter has a default value of 5 strongly supporting reads, which we established in extensive work with human datasets from large-scale disease studies, in which manual review needs to be efficient. Users will likely need to determine the optimum value for this threshold for their specific analyses. See our discussion of the AML datasets below, for suggestions on how to choose an appropriate value for this threshold.

The final two filters attempt to handle some common sources of false positives. The maximum contig overlap filter (9) helps avoid false positives due to large regions of homology between distinct genomic regions causing such regions to be incorrectly assembled together. From our experience, setting this filter to the read length, such that contigs overlapping by more than the read length are rejected, is usually appropriate. The poly(A) filter (10) helps avoid false positives when one of the alignments associated with the candidate is either a run of T’s at the very beginning of the contig or a run of A’s at the very end of the contig. The presence of poly(A) tails in assembled contigs can cause misalignments to the reference genome.

### Stage 4 Predicting chimeric transcripts

Barnacle uses the alignment topology (Stage 1.1), gene and exon-boundary annotations (Stage 1.3), and sequence properties of the candidates that have passed the user-specified filters (Stage 3) to predict chimeric transcripts of specific types. For the work described here, we focus on fusions, PTDs, and ITDs.

We predict fusion events from split-candidates with any topology. The two alignments associated with the candidate must overlap only distinct genes, and must not overlap each other in the genome (Figure 1A). Along with each fusion prediction, Barnacle reports whether or not the direction of transcription of the two genes is maintained in the contig structure. As noted above, there is no requirement that the fusion involves only canonical exon junctions.

As a PTD event, by definition, must involve at least a single full exon, we expect that such an event will usually be too long to be assembled as a full-length chimeric transcript. Instead, assembly programs like ABySS will produce a short junction contig representing the NCEJ between the copies of the duplicated exon(s). The minimum duplication length to create a junction contig rather that a full-length contig, as well as the length of the junction contigs produced, will depend on both the read and fragment lengths sequenced, and the assembly algorithm and parameters used. Such junction contigs will align as split-candidates with an eversion or duplication topology (Figure 3A). We predict PTDs from such split-candidates when both alignments associated with the candidate overlap the same gene, and both genomic breakpoint coordinates match annotated exon boundaries (Figure 1B). As noted above, these candidates may actually represent circular isoforms rather than PTDs ( [5], Figure 1D); however, as the focus of our analysis is data from poly(A)-selected cDNA libraries, we mark them as PTD events.

We predict long ITD events with criteria similar to those used for predicting PTD events, except that we require that at least one of the genomic breakpoint coordinates does not match any annotated exon boundary. However, ITDs can also be quite short, in which case assembly of the full-length chimeric transcript is possible. Short ITDs are predicted from gap-candidates that have a duplication topology (Figures 3A-v, 3C-ii, 3D-i) and at least one genomic breakpoint that matches no annotated exon boundary. The exact length threshold separating “long” ITDs, detectable as split-candidates, from “short” ITDs, detectable as gap-candidates, will depend on the assembly algorithm and parameters used.

Due to the difficulties inherent in aligning transcriptomic sequences to a genomic target sequence (the cDNA-genomic alignment problem [31]), misalignments that lead to false positive event predictions can occur. Therefore, as a final, post-processing filter, contigs representing potential fusions, PTDs, and ITDs can be aligned to wild-type transcript sequences provided by the user. Predictions involving any contig that exhibits a full-length, collinear alignment (Figure 3A-i) to any single wild-type transcript are removed from the final output.

### Stage 5 Measuring relative coverage

To support prioritizing detected events, Barnacle can estimate the coverage of a predicted chimeric transcript relative to its co-expressed wild-type transcript(s), when provided with alignments of transcriptome reads to the genome (Additional file 1: Figure S1). Because this is a fractional metric, there is no need to normalize its value when making comparisons between different genes and/or datasets.

When multiple isoforms are expressed, determining the full-length structure of a transcript with RNA-seq can be constrained by the length of the cDNA fragments produced in the sequencing experiment; that is, an RNA-seq dataset contains insufficient information to disambiguate structural options that are separated in the RNA by a distance longer than the fragment length. For example, consider a gene with five exons, in which the middle exon is longer than the fragment length. If there are reads representing transcripts both with and without the second exon, as well as reads representing transcripts both with and without the fourth exon, sequence analysis cannot determine which of the following four transcripts are actually present in the dataset: e1-e2-e3-e4-e5, e1-e3-e4-e5, e1-e2-e3-e5, and e1-e3-e5.

Given this constraint, Barnacle calculates a local metric that relies only on those portions of the transcripts for which we have direct evidence in the contig representing the chimera. For example, for a contig representing a duplication of exon 2, our method estimates the coverage of expressed chimeric transcripts that include a duplication of exon 2 and the coverage of expressed wild-type transcripts involving exon 2, then returns the value obtained by dividing the former by the latter. For a contig representing a fusion joining the first two exons of gene A (A1 and A2) to three of the last four exons of gene B (B5, B6, and B8), our method estimates the coverage of expressed chimeric transcripts that include the fusion junction between exons A2 and B5, the coverage of expressed wild-type transcripts of gene A involving exons A1 or A2, and the coverage of expressed wild-type transcripts of gene B involving exons B5, B6, or B8, then returns the two values obtained by dividing the first value by each of the latter. Cases may occur in which a gene expresses, at relatively high levels, isoforms that do not include the exon(s) that are involved in the chimera; these will reduce the accuracy of the reported relative chimeric coverage.

When a chimera involves duplication of a sequence that is longer than the read length, accurately measuring the relative coverage of that chimera requires knowing how many times the sequence is duplicated. However, given the relationship between the duplicated region length and structure, the read and fragment length, and assembly software and parameters, assembly may not provide sufficient information to determine the copy number of a duplicated region. As noted above, long duplication events will sometimes be detected through a short junction contig representing the NCEJ between the copies of the duplicated region, rather than a contig representing the full transcript (see PTD prediction in Stage 4). From such a short contig it is not possible to determine the multiplicity of the duplication. However, because we expect that lower copy number duplications are far more common than higher copy number duplications, we calculate relative coverage assuming that every duplication event involves only a single extra copy of the duplicated region.

For the read depth of the chimeric transcript, C, we use the read-to-contig support calculated by Barnacle (Stage 2 above, Figure 3E). We then define two search regions in the genome, A and B, by considering the alignment blocks of the contig-to-genome alignments. These two groups of blocks are either cut or extended so that the sum of the lengths of all blocks in each region is twice the read length (Additional file 1: Figure S1A). Each of these genomic regions is part of our attempt to determine a collection of regions that, when joined together, might represent a portion of a chimeric or wild-type transcript extending two read lengths away from the chimeric breakpoint. Using the read-to-genome alignments, we calculate the read depth, T(r,s), at each position s in each block of each search region r in {A,B}, counting only reads that overlap s by at least q nt (where q is specified by the user, and has a default value of 5). T(r,s) is made up of three values: DW(r,s) counts reads that represent sequences present only in wild-type transcripts, T_1_(r,s) counts reads that represent sequences present once in both the wild-type and chimeric transcripts, and T_2_(r,s) counts reads that represent sequences present once in wild-type transcripts, but multiple times in chimeric transcripts (Additional file 1: Figure S1B).

$$T\left(r,s\right)=\mathrm{DW}\left(r,s\right){+T}_{1}\left(r,s\right){+T}_{2}\left(r,s\right)$$

From these values, and the copy-number assumption explained above, we have the following formula for estimating the wild-type read depth at each position in regions A and B:

$$\begin{array}{l} W\left(r,s\right)=\mathrm{DW}\left(r,s\right){+I}_{1}\left(r,s\right)\times \left(T_{1}\left(r,s\right)‒C\right)\\ \;{+I}_{2}\left(r,s\right)\times \left(T_{2}\left(r,s\right)‒2\times C\right) \end{array}$$

where I_j_(r,s) = 1 if T_j_(r,s) > 0 and I_1_(r,s) = 0 otherwise, for j in {1,2}. Defining W(r) as max_s_{W(r,s)} and T(r) as max_s’_{T(r,s’) : W(r,s’) = W(r)}, we now have five values for three regions: C, the chimeric read depth at the chimeric breakpoint; W(A), the wild-type read depth in region A; T(A), the total read depth in region A; W(B), the wild-type read depth in region B; and T(B), the total read depth in region B. Two more values are calculated: W(*), the average of W(A) and W(B); and T(*), the average of T(A) and T(B). For each predicted event, Barnacle reports these seven values as well as the six ratios C/W(r) and C/T(r), for r in {A,B,*}.

For duplications, W(A) and W(B) (T(A) and T(B), respectively) represent two measures for the same gene, and we consider their average value, W(*) (T(*)) in our further analysis. For fusions, W(A) and W(B) (T(A) and T(B), respectively) represent measures of two different genes, so we consider them independently.

### Simulations

To test Barnacle’s sensitivity and specificity, we first created two simulated paired-end datasets that had distributions of read coverage comparable to the AML datasets discussed below. The first, a negative control (SIM04), contains only simulated reads generated from annotated transcript sequences. For the second, a positive control (SIM06), we simulated reads from simulated chimeric event transcripts, and combined these reads with the wild-type reads from the first dataset (see Simulation set up in Methods, below). We processed these two datasets with Trans-ABySS v1.3.5, followed by Barnacle v1.0.0. Since this is the first production-grade tool for PTD and ITD detection in RNA-seq data, we have no comparators for its performance for these event types. We compared its fusion prediction performance on these two datasets with that of TopHat-Fusion v2.0.3 (Kim and Salzberg 2011). These two datasets have 75 nt reads and mean fragment lengths of 114 nt. SIM04 comprises 38 M reads. SIM06 includes those 38 M wild-type reads as well as 3.5 M reads generated from simulated chimeric sequences, for a total of 41.5 M reads.

In the negative control dataset, SIM04, Barnacle predicts a single false positive fusion between PIGM on chromosome 1 and NCOA6 on chromosome 20, and no false positive PTDs or ITDs (Additional file 1: Table S2) shows sensitivity and false discovery rates in the positive control dataset, SIM06, for five different setups or configurations. Row 1 in this table contains values with a configuration that uses BLAT for contig-to-genome alignments and BWA for read-to-contig alignments, and has Barnacle remove predictions involving multi-mapping contigs (see Stage 3, filter 2). With this configuration, Barnacle predicts 38 PTDs, 49 ITDs and 54 fusions; 38, 46, and 52 of these predictions represent simulated PTDs, ITDs, and fusions, respectively. Below, we discuss the false positives and negatives in the positive control dataset.

Three of the 49 ITD predictions are actually misclassified PTD events, but the remaining 46 are true positives. These PTD events are misclassified because of errors made by Barnacle in determining whether the event breakpoints match annotated exon boundaries. Two of the simulated ITDs occur within exon 4 of the SGK2 gene, and because of their proximity, Barnacle groups the two contigs, each with one ITD sequence, into a single prediction (see Stage 1.2 Candidate grouping), resulting in 46 ITD predictions representing 47 simulated ITDs.

As expected, given that every read present in the negative control is also present in the positive control, one of Barnacle’s fusion predictions in SIM06 is the same false positive fusion between PIGM and NCOA6 as seen in the negative control dataset (see above, and Simulation set up in Methods, below). Another prediction is reported as a fusion between GNRH2 and SIRPA, which is not present in the simulated events; however, a fusion between GNRH2 and SIRPB1 is present in the simulated events, and there is 128 nt of exact sequence homology between SIRPA and SIRPB1 adjacent to the breakpoint of the fusion.

Of the 62 (53, 47) PTD (ITD, fusion) events that Barnacle did not predict, only 37 (30, 24) have a simulated mean coverage equal to or greater than the read-to-contig support threshold used to filter the Barnacle predictions (5 reads). Stage 1 of Barnacle identifies twelve of the 24 fusions with simulated mean coverage above our threshold, but Stage 3 filters these candidates out of the final predictions due to undercounting of read support in Stage 2. Barnacle undercounts read support in these cases because the assembled contigs are extremely short, i.e. close to or even shorter than the read length. This makes it difficult to align the reads to the contigs using BWA [32]. BWA also has trouble aligning reads to the start or end (edges) of target sequences, and some of these fusion contigs have breakpoints less than a read length from the edge. Barnacle’s fusion sensitivity improves when read support is recalculated using read-to-contig alignments generated by ABySS-map, which is capable of aligning parts of reads to short sequences and the edges of sequences (Additional file 1: Table S2, rows 2 and 4). ABySS-map is a mapping tool distributed with ABySS [33]. Note that ABySS-map only reports a single location for reads that multi-map, which can result in under-counting of read-support when multiple assembled contigs represent the same chimeric event.

Bailey et al. [34] define a segmental duplication as a region at least 1000 nt long that is duplicated within the reference genome with sequence identity greater than 90%. Five of the simulated fusion events that had coverage higher than the read-to-contig support threshold, and for which Barnacle did not undercount read support, involve genes located within segmental duplications that have 100% sequence identity. These fusions are identified as candidates in Stage 1, but are not present in the final predictions due to our use of Barnacle’s contig-to-genome multi-mapping filter (Stage 3). If we disable this filter and accept multi-mapping contigs, the fusions appear in Barnacle’s results (see Additional file 1: Table S2, rows 3, 4, and 5). However, the genes for these simulated fusions are in genomic regions whose sequences are identical over distances much longer than the 114 nt fragment length and the lengths of the genes involved in the fusions. Given this, although the assembled fusion sequence is correct, Barnacle cannot decide which of the two possible breakpoint locations is correct, and if both alignment options overlap genes, both are reported. So, for the simulated fusion between AP000351.3 and LZTR1, Barnacle reports fusions of LZTR1 with both AP000351 and KB-1125A3.10, which have nearly the same sequence. AP000351.3 and KB-1125A3.10 have two exons each; KB-1125A3.10 exon 1 is an exact subsequence of AP000351.3 exon 1 and KB-1125A3.10 exon 2 is an exact subsequence of AP000351.3 exon 2. Fortunately, for the four other simulated fusions lying in segmental duplications, only one of each of the duplicated regions contains a gene annotation, so Barnacle reports only the correct prediction. We suggest that users have Barnacle remove predictions involving multi-mapping contigs, unless specifically looking for events involving genes within known segmental duplications.

While PTD specificity is high (all 38 of the PTD predictions are true positives), PTD sensitivity is low, due in part to a BLAT limitation. As noted above in Stage 4, PTD events are often represented by short junction contigs. Accurate alignment of these contigs often involves splitting the contig into two pieces and aligning each piece of the contig to the same, or overlapping, locations in the genome. We noted that in many of these cases BLAT reports an alignment for only one of the two pieces. While GMAP [31] is better able to align such contigs, it is less effective with ITD contigs; using it improves PTD sensitivity but reduces ITD sensitivity (see Additional file 1: Table S2, row 5). Using GMAP also leads to a slight increase in fusion sensitivity, but at the cost of additional fusion false positives.

With default settings, TopHat-Fusion [24] made no fusion predictions in either simulated dataset. We suspected that this was due to the long read length and short mean fragment length used for read simulation, which correspond to the read and mean fragment lengths in the AML datasets discussed below. Since the 114 nt mean fragment length is less than twice the 75 nt read length, in most cases the pair of reads simulated from a fragment will contain overlapping sequence. Therefore, for any fragment representing the sequence across a fusion breakpoint, that breakpoint will lie within at least one of the reads generated from that fragment. This means that there will be almost no fragments with one read entirely on one side of the fusion and the other read entirely on the other (what TopHat-Fusion calls a “fusion-supporting pair”). To address this, we ran TopHat-Fusion with the supporting-pairs threshold set to 0 (the default value is 2). With this change, TopHat-Fusion predicted no fusions in SIM04, the negative control, and predicted 62 fusions in SIM06, the positive control, 60 of which are true positives (see Additional file 1: Table S2, row 6). The remaining two predictions in SIM06 (BCRP3/RTN4R and GNRH2/SIRPA) are false positives; however, they both involve genes whose sequences are extremely similar to genes involved in simulated fusions. Specifically, the part of BCRP3 involved in the predicted fusion has 97.8% sequence identity with the part of BCR that is fused to RTN4R, and the part of SIRPA involved in the predicted fusion has 99.1% sequence identity with the part of SIRPB1 that is fused to GNRH2. TopHat-Fusion predicts fusions of RTN4R with both BCR and BCRP3, but does not predict the GNRH2/SIRPB1 simulated fusion.

Although when configured with BLAT for contig-to-genome alignments and BWA for read-to-contig alignments, and removing multi-mapping contigs (Additional file 1: Table S2, row 1), Barnacle had lower fusion sensitivity than TopHat-Fusion (Additional file 1: Table S2, row 6) in our simulated dataset SIM06, using different alignment tools and allowing predictions involving multi-mapping contigs may improve Barnacle’s performance (Additional file 1: Table S2, rows 2-5). However, in our experience, removing predictions that involve multi-mapping contigs is useful in reducing false positives in real data. See also our comments below on read-support thresholds in simulated and real data.

Because the optimal aligners could differ depending on the nature of the experiment, Barnacle allows users to choose preferred read and contig alignment tools. For example, these simulations suggest that using GMAP for contig-to-genome alignments would be better for detecting PTDs, while using BLAT would be better for detecting ITDs.

Additional file 1: Table S3 gives the runtimes for the various stages of the initial configuration of pre-processing, Barnacle, and TopHat-Fusion, for the simulated dataset SIM06. Several stages of Barnacle and the Trans-ABySS pipeline take advantage of parallelization to reduce runtimes. For example, contig-to-genome alignment is split into 106 jobs, each taking only 3.02 minutes. Assuming a computer cluster capable of running 100 jobs simultaneously, Trans-ABySS pre-processing and Barnacle analysis of SIM06 can be completed in 2.42 hours. Pre-processing is dominated by the 21.6 minutes taken to sort the read-to-contig alignments. Barnacle analysis is dominated by the 55.3 minutes taken to predict events; most of this time (53.9 minutes) is spent aligning the candidate contigs to the set of wild-type transcript sequences (Stage 4). TopHat-Fusion took a total of 16.7 hours to analyse the same dataset.

We assessed the behaviour of Barnacle (with BLAT and BWA, and removing multi-mapping contigs) at read-support thresholds ranging from 1 to 200, using simulated dataset SIM06. Additional file 1: Table S4, reports the sensitivities and false discovery rates (FDRs), and Additional file 1: Figure S5 shows these as an ROC-like curve. We use FDR rather than sensitivity because the number of true negatives is not well defined for this type of experiment. With the ABySS parameters used, assembling a sequence requires a minimum local coverage of 2 reads; given this, we plot values of TPR’, which is the fraction of simulated events with simulated mean coverage at least 2 that are correctly predicted. While a read-support threshold of 1 read produces the best TPR’ and FDR for all event types for this simulated data, in our experience, such a threshold tends to produce an impractically large number of predictions in real datasets. See the discussion below of the previously published breast cancer dataset BT-474, and our suggestions for choosing an appropriate read-support threshold for the AML datasets that we analysed.

The ROC-like curves are quite different for the three event types. For PTDs, Barnacle makes no false positive predictions, even with a threshold of 1 read, so increasing the threshold merely removes weakly expressed true positives. For ITDs, the FDR increases as the read-support threshold increases, but the number of false positives does not increase. Barnacle’s three false positive ITD predictions correspond to simulated PTD events that it has misclassified as ITDs. Because these PTDs have relatively high mean simulated coverage levels, increasing the read-support threshold does not remove these false positives, until after it has removed many weakly expressed true positives. The ITD FDR decreases when going from a threshold of 50 reads to a threshold of 100 reads, because of the removal of a false positive ITD that is supported by 87 reads, causing a ‘zig-zag’ shape in the curve. For fusion predictions, with read-support thresholds of 10 or lower, the curve for fusion predictions is similar to that for ITD predictions; with read-support threshold higher than 10, it is similar to that for PTD predictions. 13 and 17 reads support the two false positive fusion predictions made by Barnacle, so when the read-support threshold is 20 or above, these two predictions are removed, resulting in an FDR of 0.

We note that while the TPR’ and FDR of Barnacle with a read-support threshold of 1 are identical to those of TopHat-Fusion, the predictions are not (Additional file 1: Table S6, Additional file 2). Each tool predicts 60 fusions; 13 of the fusions predicted by Barnacle are not predicted by TopHat-Fusion, and 13 others are predicted by TopHat-Fusion but not by Barnacle. Six of the TopHat-Fusion-specific fusions are represented by Trans-ABySS contigs that are very short, causing problems with the read-to-contig and contig-to-genome alignments; four more are represented by Trans-ABySS contigs that multi-map when aligned to the genome. One of the TopHat-Fusion-specific fusions is represented by a Trans-ABySS contig that has issues being aligned to the genome due to sequence homology between the regions flanking the fusion breakpoints. Another one of the TopHat-Fusion-specific fusions is represented by a Trans-ABySS contig that represents two distinct fusion transcripts assembled together, leading to a complex contig-to-genome alignment. The final TopHat-Fusion-specific fusion has a simulated mean coverage of only 2.86.

We assessed the effect on Barnacle’s performance of varying read and mean fragment lengths with two additional simulated datasets. SIM07 and SIM08 were created analogously to SIM06, and used the same simulated chimeric transcript sequences. SIM07 had 50 nt reads, a 150 nt mean fragment length, and 43.6 M reads; SIM08 had 75 nt reads, a 200 nt mean fragment length, and 41.4 M reads. Additional file 1: Table S7 shows that Barnacle’s performance is relatively insensitive to changes in read and mean fragment lengths; in particular, it does not depend on the mean fragment length being less than twice the read length, as it is in SIM06.

### Acute myeloid leukemia

We used Barnacle to process two poly(A)-selected acute myeloid leukemia (AML) RNA-seq datasets, A08823 and A08878, for which we sequenced 155 and 227 M read pairs, respectively. These datasets have mean fragment lengths (114 nt and 140 nt, respectively) that are shorter than twice the read length (75 nt), meaning that most pairs of reads in these datasets will overlap at their 3-prime ends. As noted above, having a mean fragment length shorter than twice the read length can challenge fusion detection tools that rely on fragments spanning fusion breakpoints with one read mapping entirely to each gene involved in the fusion. Since Barnacle does not rely on mapping reads to the reference genome for event detection, short fragment lengths are not a concern.

Prior to processing these datasets, we obtained cytogenetic evidence that the sample associated with dataset A08823 has a t(15;17) reciprocal translocation causing fusions between the promyelocytic leukemia (PML) gene on chromosome 15 and the alpha retinoic acid receptor (RARA) gene on chromosome 17. The presence of these fusions indicates the acute promyelocytic leukemia (APL) subtype of AML, which is particularly sensitive to treatment with all-trans retinoic acid [2]. Fluorescence in situ hybridization (FISH) showed a normal karyotype in dataset A08878. We also found evidence in both datasets of ITDs in the fms-related tyrosine kinase 3 (FLT3) gene by PCR amplification of the region surrounding FLT3 exon 14 (see Additional file 1: Table S8 for primer sequences), followed by size-estimation of the observed PCR bands. The presence of this ITD is associated with poor prognosis in AML [4]. Since the two AML samples we used were poly(A)-selected, and circular transcripts are not polyadenylated [5], we assume that all NCEJs predicted by Barnacle represent linear transcripts (either PTDs or shuffled exons).

As with the simulated datasets, we used Trans-ABySS to assemble our reads and perform the required alignments prior to Barnacle analysis. Initially, we filtered our predictions with the repeat filter enabled and the default read-to-contig threshold of 5 reads (see Stage 3, filters 4 and 8). See Additional file 1: Section S9 for the Barnacle commands used and Additional file 1: Table S10 for runtimes and computational resources. For these deeply-sequenced datasets the overall runtimes, including Trans-ABySS pre-processing, were ~65 hr with 100 CPUs and ~39 hr with 500 CPUs. As in the simulations, runtimes are dominated by sorting the read-to-contig alignments during preprocessing, which took 19 and 13 hr respectively. The Barnacle runtimes were 10-14 hr with 100 CPUs, and 5-7 hr on 500 CPUs.

**Figure 4:**
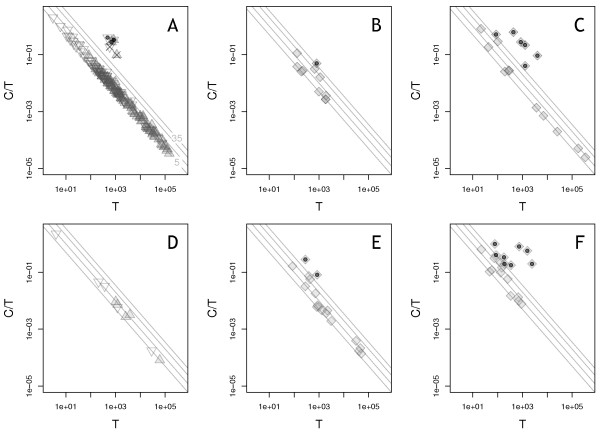
**Relative coverage of predicted chimeric transcripts in AML datasets.** Graphs show the ratio of (C)himeric read depth to (T)otal read depth as a function of (T)otal read depth, where T is the sum of read depth due to chimeric and wild-type transcripts. Dots indicate predictions that pass manual review and validation; crosses indicate predictions that fail manual review (see text). Lines indicate chimeric read-to-contig support levels of 5, 10, 20, and 35. **A)** Fusion predictions in A08823. Each fusion is represented by a triangle pointing down that uses the minimum value of T from the two genes involved in the fusion, and a triangle pointing up that uses the maximum. **B)** PTD predictions in A08823. **C)** ITD predictions in A08823. **D)** Fusion predictions in A08878. Triangle directions are as in (A). **E)** PTD predictions in A08878. **F)** ITD predictions in A08878.

After generating read-to-genome alignments using JAGuaR [35] and the hg19 human genome reference sequence, we used Barnacle to estimate the chimera-to-wild-type relative coverage of each prediction (see Stage 5, and Additional file 1: Section S11 for commands used). Figure 4 shows the relationship between the fraction of coverage attributable to the chimeric transcript, and the local total coverage, i.e. the sum of the chimeric and wild-type coverage. Using log-log axes, the majority of the predictions are close to a straight line representing our read-support threshold of 5 reads; however, there are a few outliers above and to the right of this line. Since these two datasets are so deeply sequenced, we filtered the predictions again, increasing the read-to-contig threshold to 35 reads to focus on the most confident predictions (Figure 4, other filter settings were unchanged). In our experience, plotting Barnacle relative-expression results and identifying outliers in this way is useful in selecting an appropriate read-support threshold (see Stage 2).

After this second filtering, we have 1 (2) PTD, 6 (8) ITD, and 3 (0) fusion predictions in A08823 (A08878, respectively) (see Table 1). Of the three fusion predictions in A08823, two are between PML and RARA: one joining the 5′-end of PML, ending at exon 3, to the 3′-end of RARA, starting at exon 3; the other joining the 5′-end of RARA, ending at exon 2, to the 3′-end of PML, starting at exon 4. These are the expected results of the reciprocal translocation detected by cytogenetic analysis. Manual inspection reveals that the third prediction is a false positive caused by high sequence identity between the TMEM14B and TMEM14C genes.

**Table 1:** Barnacle predictions in AML datasets A08823 and A08878

	**Type**	**Gene(s)**	**Exon(s)**^**1**^	**Read support**^**2**^	**Pred. in**	**Val.**^**3**^	**Relative Coverage**^**4**^
1	fusion	PML/RARA	e3/e3	192	A08823	WGS	27.8%/40.0%
2	fusion	RARA/PML	e2/e4	276	A08823	WGS	33.1%/33.2%
3	fusion	TMEM14B/TMEM14C	3′-utr/3′-utr	110	A08823	Failed MI	18.8%/9.8%
4	PTD	MLL	e3-e6	80	A08878	WGS	28.2%
5	PTD	SEC62	e3-e7	40 / 69	both	No WGS, RT-PCR	5.0%/8.2%^5^
6	ITD	ACADVL	e1	236	A08823	WGS	27.7%
7	ITD	ACIN1	e6	259 / 655	both	WGS	61.9%/80.4%^5^
8	ITD	AKAP2	e2	61	A08878	WGS	33.6%
9	ITD	DNHD1	e21	76	A08878	WGS	99.1%
10	ITD	FLT3^6^	e14	268	A08823	WGS	21.8%
11	ITD	FLT3^6^	e14	950	A08878	WGS	19.6%
12	ITD	FOXP1	3′-utr	64	A08878	WGS	19.6%
13	ITD	HSPBP1	e3	56	A08878	WGS	17.8%
14	ITD	KIAA1211	e8	44	A08823	WGS	51.3%
15	ITD	MRPS34	e1,i1	52	A08823	WGS	40.5%
16	ITD	PIEZO1	e32	620	A08878	WGS	57.0%
17	ITD	SND1	e1	370	A08823	WGS	9.3%
18	ITD	SSPO	e74	35	A08878	WGS	40.3%

^1^ Exon numbers are from hg19 UCSC gene annotations.

^2^ For the two chimeras predicted in both datasets, read support is presented as A08823 support / A08878 support.

^3^ Validation. WGS: validated via whole-genome shotgun sequencing. RT-PCR: validated via RT-PCR. Failed MI: failed manual inspection.

^4^ Relative coverage is presented as the local coverage attributable to the chimera, as a percent of the total local coverage (see Stage 5). For fusions, relative coverage with each parental gene is ordered as in Gene(s) column.

^5^ Relative coverage is presented as A08823 relative coverage / A08878 relative coverage.

^6^ The FLT3 duplications predicted in A08823 and A08878 have different sequences.

A 585 nt PTD involving exons 3 through 7 of SEC62 is predicted with identical sequence in both datasets, and retains the wild-type open reading frame. The other PTD prediction, a 3134 nt duplication of exons 3 through 6 of MLL, is specific to A08878, and disrupts the wild-type open reading frame. The MLL PTD is known to occur in AML patients, particularly those with a normal karyotype (such as the sample associated with A08878), and like the FLT3 ITD, is associated with poor prognosis [3].

The 14 predicted ITDs range in size from 4 nt in MRPS34 in A08823 to 48 nt in FLT3 in A08823 (see Table 2). Other than in MRPS34 and SSPO, all of the ITDs retain the wild-type open reading frame. The prediction in MRPS34 spans an exon/intron boundary and involves a retained intron adjacent to the duplication; if this intron sequence is considered, as well as the duplicated sequence, then the wild-type open reading frame is retained. Seven of the predicted ITDs (ACIN1 and KIAA1211 in A08823 and ACIN1, AKAP2, HSPBP1, PIEZO1, and SSPO in A08878) involve microrepeat expansion events (copy number increases of small three-, five-, or six-nucleotide tandem repeats present in the wild type). All of the predicted ITDs but four (in SND1 and FLT3 in A08823, and in FLT3 and SSPO in A08878) correspond to insertion records in dbSNP build 135. The FLT3 duplication predicted in A08823 has a distinct sequence from the FLT3 duplication predicted in A08878.

**Table 2:** Characterization of Barnacle ITD predictions in A08823 and A08878

	**Gene(s)**	**Exon(s)**^**1**^	**Dataset**	**Length (nt)**^**2,3**^	**Repeat Expansion?**	**Concordant dbSNP v135 ID(s)**
6	ACADVL	e1	A08823	15 (IF)	No	rs66549614, rs3835013, rs6145976
7	ACIN1	e6	both	6 (IF)	Yes	rs34293824, rs5807202, rs34870944, rs78930189,rs3077646
8	AKAP2	e2	A08878	6 (IF)	Yes	rs77728978
9	DNHD1	e21	A08878	11 + 1 (IF)	No	rs11270441, rs35685553, rs11268490, rs35369957
10	FLT3	e14	A08823	48 (IF)	No	none
11	FLT3	e14	A08878	42 + 3 (IF)	No	none
12	FOXP1	3′-utr	A08878	6 (IF)	No	rs67554413
13	HSPBP1	e3	A08878	9 (IF)	Yes	rs3040014, rs71743637, rs10701478, rs71927276
14	KIAA1211	e8	A08823	15 + 3 (IF)	Yes	rs71921617, rs11276076, rs67121617
15	MRPS34	e1,i1	A08823	4 (FS^4^)	No	rs4027362, rs33993627,rs34595082
16	PIEZO1	e32	A08878	6 (IF)	Yes	rs11281795, rs71707279
17	SND1	e1	A08823	21 (IF)	No	none
18	SSPO	e74	A08878	5 (FS)	Yes	none

^1^ Exon numbers are from hg19 UCSC gene annotations.

^2^ Length is given as either “duplication length” or “duplication length + insertion length”, when extra sequence occurs between the two copies of the duplicated sequence.

^3^ IF: in-frame, FS: frame-shift.

^4^ Event involves retention of a 152-nt intron adjacent to the duplication and is in-frame when this intron is considered as well.

FLT3 ITD events are known to include extra sequence between the two copies of the duplicated sequence in some cases [4]. Three of the ITDs that we predicted (KIAA1211 in A08823, and DNHD1 and FLT3 in A08878) include such insertions. The length of extra sequence that we observe (ranging from one, for DNHD1, to three, for FLT3 and KIAA1211, extra bases) always results in the retention of the open reading frame when considered along with the duplication.

We assessed whether there was any evidence for our predicted fusions, PTDs, or ITDs in whole-genome shotgun (WGS) sequence data for these two samples. For fusions and PTDs, we aligned the WGS reads to the hg19 human genome reference sequence using BWA v0.5.9 [32]. WGS read pairs supported both PML/RARA fusion predictions, and the MLL PTD in A08878, but not the SEC62 PTD predictions, suggesting that the latter may be the result of transcriptome-level events. To validate ITDs, we constructed wild-type and chimeric target sequences by joining together one or more copies of the duplicated sequence with 200 nt of upstream and downstream genomic sequence, then aligned the WGS reads to these target sequences using BWA v0.5.9. We found the WGS reads to support all 14 ITD predictions.

Since we found no genomic support for the SEC62 PTD predictions in either dataset, we used reverse transcription polymerase chain reaction (RT-PCR) to attempt transcriptome-level validations of this PTD (see Methods, and Additional file 1: Table S12 for primers used). Bands clearly confirmed the PTD predictions in both datasets (Additional file 1: Figure S13).

### Breast cancer

We assessed Barnacle’s fusion performance on an independent BT-474 breast cancer dataset in which two studies discovered and validated 21 gene fusions (SRA:SRP003186) [36,37]. With the default read-support threshold of 5 reads, Barnacle predicts 14 fusions (“Predictions” column in Additional file 1: Table S14). Each of the fourteen predictions corresponds to a validated fusion (“Matching” column in Additional file 1: Table S14), with 14 Barnacle predictions representing 11 validated fusion gene pairs (“Recovered” column in Additional file 1: Table S14, Additional file 1: Table S15). The differences between the “Matching” and “Recovered” columns of Additional file 1: Table S14 are due to the alternative splicing displayed by some of the validated fusions [36,37]; Barnacle treats fusion isoforms as distinct predictions, so makes multiple predictions for some of the validated fusion gene pairs.

As the number of junction reads supporting each validated fusion is reported [36,37], and eight of the validated fusions were supported by three or fewer reads, we assessed reducing Barnacle’s read support threshold (Additional file 1: Table S14). While decreasing this threshold increased the number of recovered fusions slightly, it also greatly increased the total number of predictions. For example, with a read-support threshold of 1 read, Barnacle predicted 250 fusions. Such a result set would require lengthy manual review, and so would likely be impractical in studies involving large numbers of samples. In contrast, only 4 of the predictions made with a read-support threshold of 3 do not match validated fusions. One of these predictions, which passes manual review, involves STX16 fused to GUCY1A3, while Barnacle does not predicted the validated fusion between STX16 and RAE1, which is supported by 8 junction reads. Another of the Barnacle-specific predictions is between NUMB and TPT1 (Homo sapiens tumor protein, translationally-controlled 1), which also passes manual review. The other two Barnacle-specific predictions are C3orf75/MPZL1, which from manual review looks more likely to be a potential novel ALU insertion in C3orf75 than a fusion, and APOA1BP/ZNF710, which on review is questionable due to both breakpoints being in GC-rich repetitive regions.

With a read-support threshold of 3, Barnacle recovers all but one of the validated fusions that are supported by more than 3 junction reads; the exception, discussed above, is STX16/RAE1. With a read-support threshold of 2 or 1, Barnacle recovers the validated CMTM7/GLB1 fusion that is supported by only 2 junction reads. However, as noted above, a low threshold can produce a large number of predictions for which manual review may be costly.

While the original publications validated only fusions [36,37], Additional file 1: Table S14 also reports the number of PTDs and ITDs that Barnacle predicts in BT-474 at each read-support threshold that we used.

## Conclusions

We have described Barnacle, a production-grade pipeline for detecting and characterizing chimeric transcripts in long RNA sequences, and have demonstrated its capabilities using de novo RNA-seq assembled contigs. Many methods are available for detecting fusions. In addition to fusions, Barnacle detects PTDs and ITDs in RNA-seq data; to our knowledge, it is the only method that is being used in large-scale disease studies for detecting tandem duplications [38]. It characterizes these predictions in the context of existing gene and repeat annotations; determines the level of read support; and provides metrics for prioritizing detected events with measures of coverage levels of the chimeras detected, relative to their corresponding wild-type transcripts.

Because its first stage considers a wide range of contig alignment topologies, Barnacle can be extended to identify other chimera types that are important in disease. For example, repeat expansions play a role in several diseases [39]. While Barnacle currently classifies repeat expansions as ITDs, it could be adapted to specifically characterize such events.

Although de novo assembly-based fusion detection methods are more computationally intensive than those based on read alignments to a reference genome, runtimes are practical, and de novo assembly supports characterizing detected events by generating long contig sequences that contain the exact breakpoint and its sequence context. From such contigs, validation primers can be designed, even when the contig represents a complex set of rearrangements (e.g. combining duplications, deletions, and inversions).

In two AML datasets, all ITDs and fusions, and all but one of the PTDs that passed manual inspection were validated in genomic data. The SEC62 PTD predicted in both datasets passed RT-PCR validation, but has no evidence in the genomic data, suggesting that transcriptome-level processes may have caused this chimera, consistent with fusions reported by Kannan et al. [14].

Houseley and Tollervey [40] showed that template switching in *in vitro* reverse transcription reproducibly mimics trans-splicing. This must be kept in mind when validating *in silico* chimeric transcript predictions. They also found that producing a given reverse transcriptase artifact depends on using a specific reverse transcriptase. As they recommended, we chose a different reverse transcriptase (Roche Transcriptor) for the SEC62 validations than was used in the initial sequencing (SuperScript II). Since the particular reverse transcriptase artifacts that may be produced by the SuperScript II enzyme will likely be different from those produced by the Roche Transcriptor enzyme, this reduces the chances of false validation due to such artifacts.

Given that reverse transcriptase artifacts often involve non-canonical splice sites and regions of sequence homology [40], each Barnacle prediction reports these two features, allowing the user to further evaluate whether a prediction may represent a reverse transcriptase artifact. The SEC62 PTD that we validated with RT-PCR involved only canonical splice sites, and did not involve regions of significant sequence homology.

Our simulations show that, with appropriate filter settings, Barnacle makes highly specific predictions for three types of chimeric transcripts that are important in a range of cancers: PTDs, ITDs, and fusions. High specificity makes manual review and validation efficient, which is necessary in large-scale disease studies. In AML, MLL PTDs, FLT3 ITDs, and PML/RARA fusions are important for determining prognosis, and we demonstrated Barnacle’s potential for large-scale studies by successfully predicting these events in two RNA-seq datasets. Characterizing an extended range of chimera types will help generate insights into progression, treatment, and outcomes for complex diseases.

## Methods

### Barnacle analysis pipeline

Detection and characterization of chimeric transcripts with Barnacle is a four-stage process, followed by an optional fifth stage for calculating the relative expression of chimeric transcripts relative to their corresponding wild-type transcripts. For details see Results, above.

### Simulation set up

The Barnacle package includes two tools for simulating RNA-seq experiments: event_simulator and read_simulator. The event_simulator tool simulates fusion, PTD, and ITD transcripts, and uses annotation and sequence files to create the simulated event sequences (see Additional file 1: Section S16 for details). The read_simulator tool acts as a wrapper around dwgsim, which is a whole genome next-generation sequencing simulator [41].

We used event_simulator to simulate 100 fusions, 100 PTDs, and 100 ITDs using Ensembl v59 gene annotations and the GRCh37-lite (hg19) human genome reference sequence, restricting our simulations to genes on chromosomes 20 and 22 (see Additional file 1: Section S17 for the parameters used, see Additional file 2 for the simulated events). We removed any simulated transcript sequence less than 200 nt long, leaving us with a total of 99 simulated fusions, 100 simulated PTDs, and 100 simulated ITDs. We used an in-house paired-end RNA-seq read-to-genome alignment analysis pipeline (described in [20]) on one of our real datasets, A08823, to estimate the coverage to simulate for our wild-type and event sequences. This pipeline uses BWA [32] for alignment generation. For each wild-type sequence from chromosome 20 or chromosome 22, we used read_simulator to simulate per-gene mean coverage values equal to those measured in A08823, generating 38 million read pairs from Ensembl v59 transcript sequences. We also used read_simulator to simulate a total of 3.5 million read pairs from our simulated event sequences (see Additional file 1: Section S18 for the read_simulator parameters used), using coverage values sampled from a model consisting of two overlapped log-normal distributions, whose parameters were selected to closely match the coverage distribution of A08823 (see Additional file 1: Section S19, Additional file 1: Figure S20). The mean read coverage of our event sequences ranges from 0.1285 to 2135, with a median of 33.15, a mean of 123.3 and a standard deviation of 245.7. After generating reads, 74 simulated PTDs, 77 ITDs, and 76 fusions have mean read coverage values greater than 5 reads, Barnacle’s default read-support threshold (see Stage 3, filter 8). The reads from the wild-type sequences were used for our negative control dataset, SIM04. The reads from the wild-type sequences were combined with the reads from our simulated event sequences to create our positive control dataset, SIM06.

We used Trans-ABySS to assemble the simulated datasets and create the contig-to-genome and read-to-contig alignment files that Barnacle requires (see Results and Discussion above for Barnacle input files). To generate contigs representing (simulated) transcripts having a wide range of expression levels, Trans-ABySS performed multiple assemblies with appropriate parameter settings, and then merged the resulting contig sets into a meta-assembly of non-redundant contigs. Trans-ABySS then aligned these contigs to the hg19 human genome reference sequence using BLAT [29]. The Trans-ABySS pipeline also served as a wrapper around BWA [32] to align the input reads to the assembled contigs. We ran Barnacle on the Trans-ABySS-assembled contigs and contig-to-genome and read-to-contig alignments for each dataset (see Additional file 1: Section S21 for the commands used).

We also processed these two datasets using TopHat-Fusion v2.0.3 [24] (see Additional file 1: Section S22 for the commands used).

### Library construction and sequencing

Total RNA samples (2-3 μg) were arrayed into a 96-well plate and polyadenylated (polyA+) mRNA was purified using a 96-well MultiMACS mRNA isolation kit on a MultiMACS 96 separator (Miltenyi Biotec, Germany) with on column DNaseI-treatment as per the manufacturer’s instructions. Eluted polyA + RNA was ethanol precipitated and resuspended in 10 μL of DEPC treated water with 1:20 SuperaseIN (Life Technologies, USA). Double-stranded cDNA was synthesized from the purified polyA + RNA using the Superscript Double-Stranded cDNA Synthesis kit (Life Technologies, USA) and random hexamer primers at a concentration of 5 μM. The cDNA was quantified in a 96-well format using PicoGreen (Life Technologies, USA) and VICTOR3V Spectrophotometer (PerkinElmer, Inc. USA). The quality was checked on a random sampling on the Agilent using the High Sensitivity DNA chip Assay. We fragmented cDNA by Covaris E210 (Covaris, USA) for 55 seconds, a “Duty cycle” of 20% and “Intensity” of 5. Plate-based libraries were prepared following the BC Cancer Agency, Genome Sciences Centre paired-end (PE) protocol on a Biomek FX robot (Beckman-Coulter, USA). Briefly, the cDNA was purified in 96-well format using Ampure XP SPRI beads, and was subject to end-repair and phosphorylation by T4 DNA polymerase, Klenow DNA polymerase, and T4 polynucleotide kinase respectively in a single reaction, followed by cleanup using Ampure XP SPRI beads and 3′ A-tailing by Klenow fragment (3′ to 5′ exo minus). After cleanup using Ampure XP SPRI beads, PicoGreen quantification was performed to determine the amount of Illumina PE adapters used in the next step of adapter ligation reaction. The adapter-ligated products were purified using Ampure XP SPRI beads, then PCR-amplified with Phusion DNA polymerase (Thermo Fisher Scientific Inc. USA) using Illumina’s PE primer set, with cycle conditions: 98˚C for 30 sec followed by 10 cycles of 98˚C for 10 sec, 65˚C for 30 sec and 72˚C for 30 sec, and then 72˚C for 5 min. The PCR products were purified using Ampure XP SPRI beads, and checked with Caliper LabChip GX for DNA samples using the High Sensitivity Assay (PerkinElmer, Inc. USA). PCR product of desired size range was purified using an in-house 96-channel size-selection robot, and the DNA quality was assessed and quantified using an Agilent DNA 1000 series II assay and Quant-iT dsDNA HS Assay Kit using Qubit fluorometer (Invitrogen), then diluted to 8 nM. The final concentration was verified by Quant-iT dsDNA HS Assay prior to Illumina HiSeq2000 PE 75 base sequencing.

### De novo assembly and processing

For each dataset, transcriptome assemblies were performed using Trans-ABySS v1.3.5 as previously described in Robertson et al. [20], with the following modifications. After the assembly-merging stage, all the original read pairs were aligned to the merged contig set using BWA v0.5.9 [32] and converted to BAM format using SAMtools v0.1.18 [42]. The resulting contigs were aligned to the GRCh37-lite (hg19) human genome reference sequence using BLAT v34 [29].

### Annotation files

In our simulation experiments we used Ensembl v59 annotations and sequences for simulating events and Ensembl v65 annotations and sequences for the Barnacle gene and exon coordinate annotations and the TopHat-Fusion analysis. To process the AML datasets we used the hg19 UCSC gene annotations and transcript sequences, downloaded from UCSC in February 2012 [43], for the Barnacle gene and exon coordinate annotations. In both analyses, we used the hg19 RepeatMasker [44] and SimpleRepeats/Tandem Repeats Finder [45] annotations, downloaded from UCSC in February 2012, for the Barnacle repeat and small structural RNA annotations. We used Ensembl v59 annotations for JAGuaR processing of AML RNA-seq data.

### RT-PCR validation

Primers were designed with Primer3 and supplied by Integrated DNA Technologies (Coralville, Iowa). First-strand cDNA was synthesized using 1 μg of total RNA, following the Roche Transcriptor First Strand cDNA Synthesis protocol (Catalog #04896866001). 2 μL of the 2.5-fold diluted template is used for setting up the PCR reaction in 48 μl: 2 μl template, 39.4 μl Nuclease-free water, 2 μl 50-mM Magnesium sulphate, 0.4 μl 25-mM dNTPs, 5 μl 10x High Fidelity Buffer, 0.5 μl 20-μM Forward Primer, 0.5 μl 20-μM Reverse Primer, and 0.2 μl Platinum High-Fidelity DNA polymerase. PCR was run with 94˚C for 2 min, followed by 36 cycles of 94˚C for 15 sec, 60˚C for 15 sec, 68˚C for 15 sec, and then 68˚C for 10 min. Three-quarters of the PCR product was run on 3% agarose gel with 0.08% ethidium bromide for 45 min at 150 V.

### Data access

#### Data

Reads, assembled contigs, contig-to-genome alignments, read-to-genome alignments, and analysis files from the simulations are bundled with the Barnacle software distribution.

RNA-seq and read-to-genome alignments for A08823 and A08878 are available at the Short Read Archive as study accession SRP015761.

Assembled contigs, contig-to-genome alignments, and Barnacle analysis files for A08823 and A08878 are available at [46].

#### Software

Current and previous versions of Barnacle are available at: [46].

## Abbreviations

AML: Acute myeloid leukemia; cDNA: complementary DNA (DNA synthesized from mRNA); chr: chromosome; CPU: Central processing unit; dbSNP: the Single Nucleotide Polymorphism database; DEPC: Diethylpyrocarbonate (an RNase inhibitor); DNA: Deoxyribonucleic acid; DNase: Deoxyribonuclease; FDR: False discovery rate; FISH: Fluorescence In Situ Hybridization; FS: Frame-shift; IF: In-Frame; ITD: Internal tandem duplication; mRNA: messenger RNA; NCBI: National center for biotechnology information; NCEJ: Non-canonical exon junction; nt: nucleotides; PTD: Partial tandem duplication; PTES: Post-transcriptional exon shuffling; RNA: Ribonucleic acid; RNA-seq: high-throughput RNA sequencing; RNase: Ribonuclease; RT-PCR: Reverse transcription polymerase chain reaction; snRNA: small nuclear RNA; TPR: True positive rate; tRNA: transfer RNA; UCSC: University of California, Santa Cruz; Utr: untranslated region; WGS: Whole genome shotgun sequencing.

## Competing interests

Barnacle results are part of the contributions made by the Genome Sciences Centre to a manuscript (in press) on a Cancer Genome Atlas study on acute myeloid leukemia. There are no related manuscripts in press or submitted. The material in this manuscript was used in LS’s Master of Science thesis in the Department of Computing Science at Simon Fraser University in Vancouver, BC. The authors declare that they have no competing interests.

## Authors’ contributions

LS developed and applied Barnacle and drafted the manuscript. GR participated in analysis and helped draft the manuscript. KLM participated in analysis and manual review of results. YSB, RDC, TRD, and NT developed and applied read-alignment methods. RC, SDJ, KMN, JQQ, and AR developed assembly and assembly analysis methods, generated assemblies, and participated in analysis. DH performed PCR identification of FLT3 ITD events. RAM was responsible for sequencing. AJM, JP, and SS validated predicted chimeric events. AT and YJZ constructed libraries. RV supported data management. SW was responsible for project coordination. DY contributed to Barnacle development. PAH and AK supervised scientific direction. SCS supervised method development. IB supervised scientific direction, method development, data analysis, and drafting of the manuscript. All authors read and approved the final manuscript.

## Supplementary Material

Additional file 1 **Supplement for Barnacle: detecting and characterizing tandem duplications and fusions in transcriptome assemblies.** This file contains supplementary figures, tables, and text. Click here for file

Additional file 2 Simulated events and analysis results with Barnacle and TopHat-Fusion. Click here for file
