# Supplementary material for: Barnacle: detecting and characterizing tandem duplications and fusions in transcriptome assemblies
Source: BMC Genomics. 2013 Aug 14;14:550. doi: 10.1186/1471-2164-14-550 (PMC3751903; doi:10.1186/1471-2164-14-550)
Supplement: Additional file 1 — Supplement for Barnacle: detecting and characterizing tandem duplications and fusions in transcriptome assemblies. This file contains supplementary figures, tables, and text. [file 1471-2164-14-550-S1.pdf]

Supplement for

Barnacle: detecting and characterizing tandem duplications and fusions in transcriptome assemblies

Lucas Swanson[1,2], Gordon Robertson[1], Karen L. Mungall[1], Yaron S. Butterfield[1], Readman Chiu[1], Richard D. Corbett[1], T. Roderick Docking[1], Donna Hogge[3], Shaun D. Jackman[1], Richard A. Moore[1], Andrew J. Mungall[1], Ka Ming Nip[1], Jeremy Parker[1], Jenny Q. Qian[1], Anthony Raymond[1], Sandy Sung[1], Angela Tam[1], Nina Thiessen[1], Richard Varhol[1], Sherry Wang[1], Deniz Yorukoglu[1,2,5], YongJun Zhao[1], Pamela A. Hoodless[3,4], S. Cenk Sahinalp[2], Aly Karsan[1], Inanc Birol[1,2,4]

[1] Canada's Michael Smith Genome Sciences Centre, British Columbia Cancer Agency, Vancouver, Canada
[2] School of Computing Science, Simon Fraser University, Burnaby, Canada
[3] Terry Fox Laboratory, British Columbia Cancer Agency, Vancouver, Canada
[4] Department of Medical Genetics, University of British Columbia, Vancouver, Canada
[5] Computer Science and Artificial Intelligence Laboratory, Massachusetts Institute of Technology, Cambridge, USA

S1) Calculating relative coverage with Barnacle.

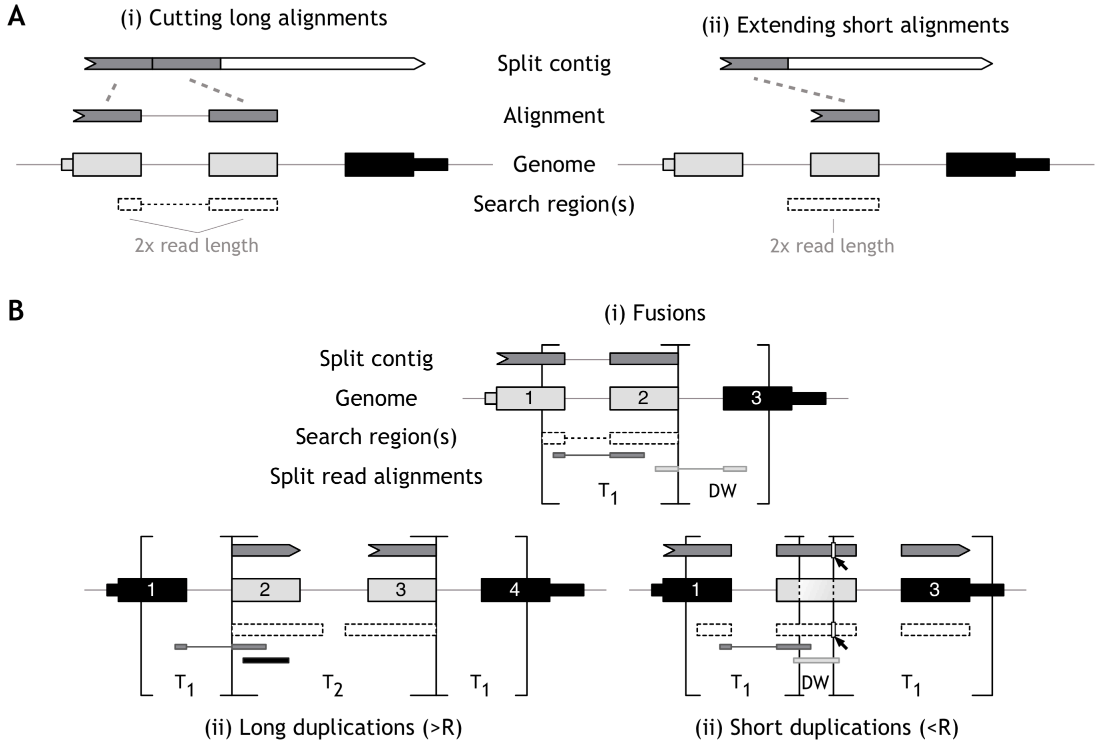

A) Barnacle uses contig-to-genome alignments to define two genomic regions to use in calculating the relative coverage of a predicted chimeric transcript. One such search region is shown, first for the case where the portion of the contig involved in the alignment to the genome is longer than twice the read length (i), and then for the case where it is shorter (ii). **B)** Reads with alignments overlapping the search region are placed into one of three groups, depending on whether they involve sequence that appears twice in the chimera (T_2), once in the chimera (T_1), or never in the chimera (DW). See **Stage 5** Measuring relative coverage for details.

S2) Barnacle and TopHat-Fusion sensitivity and false discovery rates on simulated dataset SIM06.

	PTDs			ITDs			Fusions		
	**TPR**	**TPR***	**FDR**	**TPR**	**TPR***	**FDR**	**TPR**	**TPR***	**FDR**
Barnacle + BLAT + BWA	0.380	0.514	0.000	0.470	0.610	0.060	0.526	0.684	0.037
Barnacle + BLAT + ABySS-map	0.380	0.514	0.000	0.490	0.636	0.109	0.586	0.750	0.033
Barnacle-MM + BLAT + BWA	0.380	0.514	0.000	0.500	0.649	0.107	0.576	0.750	0.050
Barnacle-MM + BLAT + ABySS-map	0.380	0.514	0.000	0.510	0.662	0.105	0.636	0.816	0.045
Barnacle-MM + GMAP + ABySS-map	0.550	0.740	0.000	0.300	0.390	0.118	0.646	0.829	0.059
TopHat-Fusion							0.606	0.690	0.032

TPR = TP / (TP + FN) is the true positive rate (sensitivity), where TP is the number of true positives and FN is the number of false negatives.

TPR* = TP* / (TP* + FN*) is the adjusted true positive rate, where TP* (FN*) is the number of true positives (false negatives) with mean simulated coverage greater than the default read-support threshold of the tool (5 for Barnacle, 3 for TopHat-Fusion).

FDR = FP / (FP + TP) is the false discovery rate, where FP is the number of false positives.

'Barnacle' represents running Barnacle with default filter settings; 'Barnacle-MM' represents running Barnacle without filtering out multi-mapping contigs.

BWA and ABySS-map denote the tool used to align reads to contig sequences.

BLAT and GMAP denote the tool used to align contig sequences to the genome.

TopHat-Fusion results use a supporting-pair threshold of 0 (see **Simulations**).

See **Simulations** in the main text for further description of table rows.

S3) Trans-ABySS, Barnacle, and TopHat-Fusion runtimes on simulated dataset SIM06 (20.8 M read pairs).

Stage	Runtime	# CPUs or jobs[1]
Trans-ABySS 1: assemblies (x19)[2]	13.7 min	12 CPUs
Trans-ABySS 2: filtering, extending, merging	8.02 min	8 CPUs
Read to contig alignment 1: BWA aln	2.80 min	12 CPUs
Read to contig alignment 2: BWA sampe, SAMtools sort	21.6 min	1 CPU
Contig to genome alignments[3]	3.02 min	106 jobs
Pre-processing total[4]	1.32 hr	
Barnacle 1: setup data and submit candidate identification jobs	1.92 min	1 CPU
Barnacle 2: candidate identification jobs[3]	37.5 sec	106 jobs
Barnacle 3: group and annotate candidates, submit read support jobs	3.20 min	1 CPU
Barnacle 4: read support jobs[3]	3.98 min	4 jobs
Barnacle 5: integrate read support	5.07 sec	1 CPU
Barnacle 6: filter candidate groups	5.92 sec	1 CPU
Barnacle 7: predict events	55.3 min	1 CPU
Barnacle total[4]	65.8 min	
TopHat (alignment)	3.97 hr	1 CPU
TopHat-Fusion (fusion calling)	12.7 hr	1 CPU
TopHat-Fusion total	16.7 hr	

[1] Use of ‘jobs’ in this column indicates that the stage is embarrassingly parallel. Each job uses 1 CPU.

[2] One assembly for each of 19 k-values, each using the indicated number of CPUs.

[3] Mean runtime per job.

[4] Assuming a computer cluster running an average of 100 jobs at once.

S4) Barnacle sensitivity and false discovery rate on simulated dataset SIM06 with varying read-support thresholds.

Read thresh.	PTDs TPR	PTDs TPR*	PTDs TPR'	PTDs FDR	ITDs TPR	ITDs TPR*	ITDs TPR'	ITDs FDR
1	0.400	0.440	0.471	0.000	0.490	0.505	0.570	0.058
2	0.390	0.447	0.459	0.000	0.490	0.570	0.570	0.058
3	0.390	0.463	0.459	0.000	0.480	0.608	0.558	0.059
4	0.390	0.494	0.459	0.000	0.480	0.608	0.558	0.059
5	0.380	0.514	0.447	0.000	0.470	0.610	0.547	0.060
10	0.340	0.472	0.400	0.000	0.420	0.646	0.488	0.067
25	0.260	0.441	0.306	0.000	0.330	0.660	0.384	0.083
50	0.200	0.513	0.235	0.000	0.280	0.718	0.326	0.098
100	0.120	0.480	0.141	0.000	0.200	0.625	0.233	0.091
200	0.080	0.500	0.094	0.000	0.140	0.650	0.163	0.125

Read thresh.	Fusions TPR	Fusions TPR*	Fusions TPR'	Fusions FDR
1	0.606	0.638	0.674	0.032
2	0.586	0.652	0.652	0.033
3	0.566	0.655	0.629	0.034
4	0.545	0.663	0.607	0.036
5	0.525	0.684	0.584	0.037
10	0.485	0.727	0.539	0.040
25	0.374	0.712	0.416	0.000
50	0.293	0.725	0.326	0.000
100	0.192	0.594	0.213	0.000
200	0.071	0.467	0.079	0.000

'Read thresh.' is the Barnacle read-support threshold used (see **Stage 2** and **Stage 3** in **Results and Discussion**).

TPR = TP / (TP + FN) is the true positive rate (sensitivity), where TP is the number of true positives and FN is the number of false negatives.

TPR* = TP* / (TP* + FN*) is the adjusted true positive rate, where TP* (FN*) is the number of true positives (false negatives) with a mean simulated coverage greater than the read-support threshold used.

TPR' = TP' / FN' is the true positive rate considering only events with a mean simulated coverage of at least 2.

FDR = FP / (FP + TP) is the false discovery rate, where FP is the number of false positives.

For the above results, BLAT was used for contig-to-genome alignments, BWA was used for read-to-contig alignments, and default Barnacle filter settings were used except for the read-support threshold, which is as indicated.

S5) ROC-like curves using TPR' and FDR for simulated dataset SIM06.

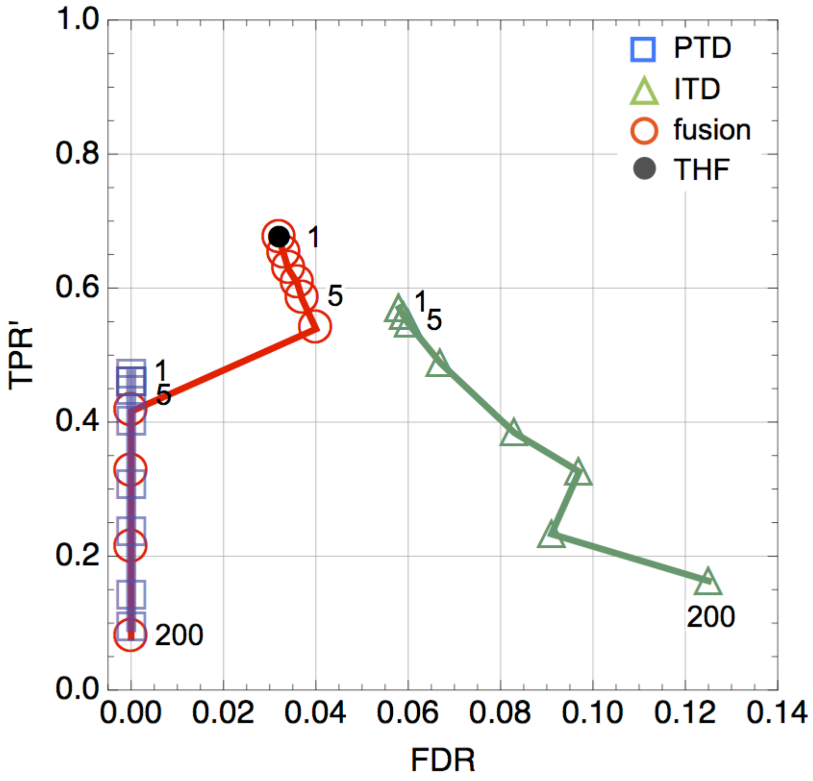

This figure uses values from S2, above, where TPR' and FDR are defined. Positions of points for read-support thresholds of 1, 5, and 200 are indicated.

S6) Simulated events, with Barnacle and TopHat-Fusion analysis results.

See accompanying spreadsheet "Swanson_Barnacle_Simulated_Events.xls".

S7) Barnacle sensitivity and false discovery rate on simulated datasets with varying read and mean fragment lengths.

Dataset	Read length	Mean fragment length	PTDs		ITDs		Fusions	
			TPR	FDR	TPR	FDR	TPR	FDR
SIM06	75	114	0.380	0.000	0.470	0.060	0.526	0.037
SIM07	50	150	0.370	0.000	0.350	0.102	0.545	0.000
SIM08	75	200	0.320	0.000	0.480	0.094	0.535	0.036

For the above results, BLAT (for contig-to-genome alignments), BWA (for read-to-contig alignments), and default Barnacle filter settings were used.

S8) FLT3 primers used prior to Barnacle analysis.

	Forward	Reverse
FLT3	GCAATTTAGGTATGAAAGCCAGC	CTTTCAGCATTTTGACGGCAACC

S9) Barnacle commands used for AML analysis.

```
~ ${barnacle_dir}/src/barnacle.pl -lib A08823 -lib_dir ${data_dir}/A08823 -config
  ${data_dir}/project.cfg -identify_candidates -cluster ${cluster_submit_hostname}
~ ${barnacle_dir}/src/utils/submit.py A08823-support
  ${data_dir}/A08823/Assembly/abyss-1.3.2/barnacle/ver_1.0.0.0/run_support.sh
  ${cluster_submit_hostname} --memory 8G
~ ${barnacle_dir}/src/support/read_to_contig/integrate.py A08823
  ${data_dir}/A08823/Assembly/abyss-1.3.2/barnacle/ver_1.0.0.0/5_breakpoint_genes/A08823.b
  arnacle.data
~ ${barnacle_dir}/src/filter/filter_groups.py A08823
  ${data_dir}/A08823/Assembly/abyss-1.3.2/barnacle/ver_1.0.0.0/6_with_r2c/A08823.barnacle.
  data --max-num-groups 3 --no-multi-mapping --no-homopolymers --no-repeats
  --no-struct-RNA --min-identity 99.0 --min-ctg-rep 0.9 --read-to-contig 35
  --max-ctg-overlap 75 --no-polyA-events
~ ${barnacle_dir}/src/prediction/predict_events.py A08823
  ${data_dir}/A08823/Assembly/abyss-1.3.2/barnacle/ver_1.0.0.0/7_filtered/A08823.barnacle.
  pass --transcript-annotations ${barnacle_dir}/annotations/UCSC_genes_ref.txt
  --transcript-sequences ${barnacle_dir}/annotations/UCSC_genes_hg19.fa --contig-sequences
  ${data_dir}/A08823/Assembly/abyss-1.3.2/barnacle/ver_1.0.0.0/1_raw_candidates/A08823.bar
  nacle.contigs
```

${barnacle_dir} represents the directory in which Barnacle is installed.
${data_dir} represents the directory to which the data was downloaded.
${cluster_submit_hostname} represents the hostname used to submit cluster jobs.
A08878 was processed with the same commands as above, replacing “A08823” with “A08878”.

S10) Trans-ABySS and Barnacle runtimes for AML datasets.

Stage	A08823 (155M read pairs) Runtime (hrs)	A08823 (155M read pairs) # CPUs or jobs[1]	A08878 (227M read pairs) Runtime (hrs)	A08878 (227M read pairs) # CPUs or jobs[1]
Trans-ABySS 1: assemblies (x19)[2]	4	12 CPUs	5	12 CPUs
Trans-ABySS 2: filtering, extending, merging	3.3	8 CPUs	7.3	8 CPUs
Read to contig alignment 1: BWA aln	4	12 CPUs	6	12 CPUs
Read to contig alignment 2: BWA sampe, SAMtools sort	19	1 CPU	13	1 CPU
Contig to genome alignments[3]	0.6	1722 jobs	0.5	2774 jobs
Pre-processing total[4]	49 (32)		57 (34)	
Barnacle 1: setup data and submit candidate identification jobs	1	1 CPU	1	1 CPU
Barnacle 2: candidate identification jobs[3]	0.015	1722 jobs	0.012	2774 jobs
Barnacle 3: group and annotate candidates, submit read support jobs	3.5	1 CPU	2.3	1 CPU
Barnacle 4: read support jobs[3]	5.5	156 jobs	4.2	140 jobs
Barnacle 5: integrate read support	0.22	1 CPU	0.13	1 CPU
Barnacle 6: filter candidate groups	0.21	1 CPU	0.17	1 CPU
Barnacle 7: predict events[5]	0.13 (0.046)	1 CPU	0.32 (0.15)	1 CPU
Barnacle total[4]	14 (6.8)		10 (5.2)	

[1] Use of "jobs" in this column indicates that the stage is embarrassingly parallel. Each job uses 1 CPU.
[2] One assembly for each of 19 k-values, each using the indicated number of CPUs.
[3] Mean runtime per job.
[4] Assuming a computer cluster running an average of 100 (500) jobs at once.
[5] Runtime for read-support threshold of 5 (35).

S11) Barnacle relative coverage commands used.

```
~  ${barnacle_dir}/src/expression/expression_estimator.py A08823
   ${data_dir}/A08823/Assembly/abyss-1.3.2/barnacle/ver_1.0.0.0/8_predicted_events/A08823.b
   arnacle.${event_type} ${data_dir}/A08823/Reads_to_genome/A08823_jaguar.bam
```

${barnacle_dir} represents the directory in which Barnacle is installed.
${event_type} is one of "fus", "fus.mix_dirs", "ptd", "itd", "itd.multi_exon", "itd.edge_gap", "itd.edge_gap.multi_exon", "itd.full_ctg_dup", or "itd.full_ctg_dup.multi_exon".
A08878 was processed with the same commands as above, replacing "A08823" with "A08878".

S12) RT-PCR validation primers for Barnacle *SEC62* PTD predictions in A08823 and A08878.

	Forward	Reverse
SEC62 e3-e4 WT	GGCCAAGAAAGGAGAGGAAG	TTTTAGGGCTCGGTGAAAAA
SEC62 e7-e3 PTD	TTACCTCAGTGTGGGTGCAG	ACCACAGACTCCCTGGTTGT

S13) RT-PCR PTD validation gel image for Barnacle *SEC62* PTD predictions in A08823 and A08878.

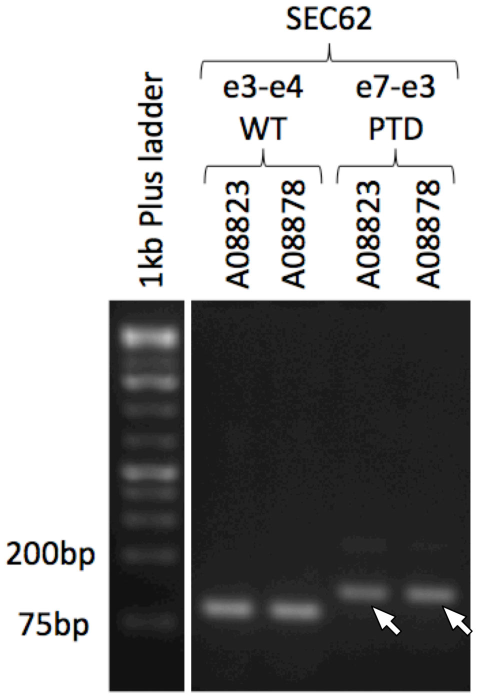

"*SEC62* e3-e4 WT" lanes serve as controls, and use primers that flank the junction from exon 3 to exon 4, which is common to both wild-type and chimeric *SEC62* transcripts. "*SEC62* e7-e3 PTD" lanes use primers that flank the junction from exon 7 to exon 3, which is only present in chimeric *SEC62* transcripts.

S14) Barnacle analysis of the previously published breast cancer dataset BT-474 [36, 37].

Read thresh.	PTDs	ITDs	Predictions	Fusions Matching	Recovered
5	1	6	14	14	11
3	3	16	19	15	12
2	7	24	34	17	13
1	10	48	250	17	13

‘Predictions’ is the number of fusion predictions that Barnacle made.
‘Matching’ is the number of fusion predictions that represent validated fusions.
‘Recovered’ is the number of validated fusions that are represented by fusion predictions.
See text for an explanation of why ‘Recovered’ is less than ‘Matching’, and for a discussion of missed fusions.

S15) Fusions validated in BT-474 [36, 37].

Gene Pair	Junction Reads	Detected by Barnacle with read-support threshold: 5	3	2	1
DIDO1/KIAA0406	1	No	No	No	No
CMTM7/GLB1	2	No	No	Yes	Yes
CPNE1/PI3	2	No	No	No	No
BCAS3/MED13	3	No	No	No	No
MRPL45/TRPC4AP	3	No	No	No	No
MED1/USP32	3	No	No	No	No
PIP4K2B/RAD51C	3	No	No	No	No
LAMP1/MCF2L	3	No	No	No	No
DOK5/STARD3	6	No	Yes	Yes	Yes
MYO19/SKA2	7	Yes	Yes	Yes	Yes
RAE1/STX16	8	No	No	No	No
ACSF2/MED1	10	Yes	Yes	Yes	Yes
AHCTF1/NAAA	11	Yes	Yes	Yes	Yes
MYO9B/RAB22A	12	Yes	Yes	Yes	Yes
MED1/STXBP4	13	Yes	Yes	Yes	Yes
CEP250/ZMYND8	14	Yes	Yes	Yes	Yes
IKZF3/VAPB	26	Yes	Yes	Yes	Yes
AC090627.1/THRA	38	Yes	Yes	Yes	Yes
SYNRG/TOB1	38	Yes	Yes	Yes	Yes
RPS6KB1/SNF8	68	Yes	Yes	Yes	Yes
ACACA/STAC2	72	Yes	Yes	Yes	Yes

S16) Event simulation with Barnacle.

The following pseudocode describes Barnacle's event sequence simulation. All randomization uses the random module in Python 2.6.

```
SimulateFusion
Select chrA uniformly at random
Choose interchromosomal or intrachromosomal with user specified threshold
If interchromosomal, select chrB uniformly at random (excluding chrA)
If intrachromosomal, set chrB equal to chrA
Select geneA from chrA uniformly at random
Select geneB from chrB uniformly at random
Choose whether to force breakpoints to occur at exon edges with user specified threshold
If forcing breakpoints to occur at exon edges, select one exon from each gene uniformly
   at random and use the end of the exon selected for geneA as positionA and beginning of
   the exon selected for geneB as positionB
If not forcing breakpoints to occur at exon edges, select positionA and positionB from
   geneA and geneB, respectively, uniformly at random
Use the sequence of geneA up to positionA and the sequence of geneB after positionB
Choose standard or mixed-sense with user specified threshold
If mixed-sense, choose either geneA or geneB with equal probability and reverse the
   portion of the simulated fusion transcript sequence coming from the chosen gene

SimulatePTD
Select a gene uniformly at random
// The selected exon(s) must be "internal" to the gene (i.e. not the first or last exon
   of the gene), because both sides of the duplicated region must match up with exon
   boundaries involved in splicing (by the definition of a PTD)
Select internal exonA uniformly at random
Choose single exon or multiple exons with user specified threshold
If simulating a single exon PTD, set exonB equal to exonA
If simulating a multiple exon PTD, select internal exonB uniformly at random
Set positionA to min(exonA.start, exonB.start)
Set positionB to max(exonA.end, exonB.end)
Duplicate the sequence of the selected gene between positionA and positionB
Choose whether to add extra sequence between the copies of the duplicated region with
   user specified threshold
If adding extra sequence, randomly select the length of the extra sequence (with user
   specified minimum and maximum length), then select the required number of bases
   uniformly at random and insert them between the copies of the duplicated region

SimulateITD
Select a gene uniformly at random
Select an exon from that gene (with UTRs trimmed off) uniformly at random
Select positionA and positionB within that exon uniformly at random, ensuring that the
   distance between positionA and positionB is within user specified thresholds and that
   the positions between positionA and positionB do not all contain the same base
Duplicate the sequence of the selected gene between positionA and positionB
Choose whether to add extra sequence between the copies of the duplicated region with
   user specified threshold
If adding extra sequence, randomly select the length of the extra sequence (with user
   specified minimum and maximum length), then select the required number of bases
   uniformly at random and insert them between the copies of the duplicated region
```

S17) Commands used to simulate chimeric transcript sequences with event_simulator.

```
~  ${barnacle_dir}/src/utils/simulator/event_simulator.py
  ${barnacle_dir}/annotations/ensembl59/Homo_sapiens.GRCh37.59.gtf
  ${barnacle_dir}/annotations/hg19.2bit
  ${barnacle_dir}/sample_data/SIM05/simulated_transcripts --num-fusions 100 --num-ptds 100
  --num-itds 100 --chr-filter 20,22 --min-len 200 --seed 1340122071
```

${barnacle_dir} represents the directory in which Barnacle is installed.

S18) Commands used to simulate read sequences with read_simulator.

```
~  ${barnacle_dir}/src/utils/simulator/read_simulator.py
  ${barnacle_dir}/annotations/ensembl59/Homo_sapiens.GRCh37.59.gtf
  ${barnacle_dir}/sample_data/SIM04/wildtype_transcripts.fa
  ${barnacle_dir}/sample_data/SIM04/wildtype_coverage.tsv
  ${barnacle_dir}/sample_data/SIM05/simulated_transcripts.fa
  ${barnacle_dir}/sample_data/SIM05/simulated_coverage.txt -F 114 --std-dev 11.9
  --chr-filter 20,22 --seed 1340656577
```

${barnacle_dir} represents the directory in which Barnacle is installed.

S19) Commands used to simulate coverage distribution (Mathematica v8).

```
covsLnA=RandomVariate[LogNormalDistribution[4.8,1.18],10200*3];
covsLnB=RandomVariate[LogNormalDistribution[0.85,2.2],18200*3];
covsLnMix=Join[covsLnA,covsLnB];
shuffled=RandomSample[covsLnMix,83000];
```

S20) Histograms of coverage distributions used for chimeric transcript simulation.

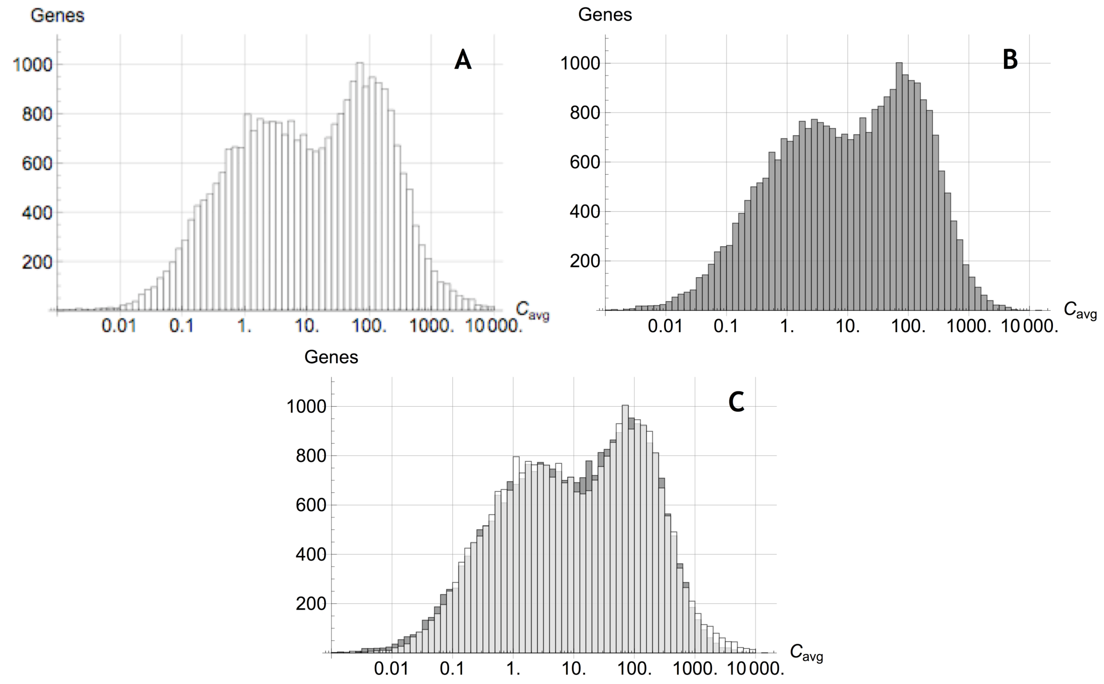

A) A08823 coverage distribution. **B)** Simulated coverage distribution. **C)** Overlap of A08823 and simulated coverage distributions. C_{avg} is the average exonic read coverage or depth.

S21) Barnacle commands used for Barnacle analysis on simulated data (for commands used to generate simulated data see **Table S1, S2**).

```
~  ${barnacle_dir}/src/barnacle.pl -lib SIM04 -lib_dir ${barnacle_dir}/sample_data/SIM04
 -config ${barnacle_dir}/sample_data/sample.cfg -identify_candidates -cluster
 ${cluster_submit_hostname}
~  ${barnacle_dir}/src/utils/submit.py SIM04-support
 ${barnacle_dir}/sample_data/SIM04/Assembly/abyss-1.3.2/barnacle/ver_1.0.0.0/run_support.
 sh ${cluster_submit_hostname} --memory 8G
~  ${barnacle_dir}/src/support/read_to_contig/integrate.py SIM04
 ${barnacle_dir}/sample_data/SIM04/Assembly/abyss-1.3.2/barnacle/ver_1.0.0.0/5_breakpoint
 _genes/SIM04.barnacle.data
~  ${barnacle_dir}/src/filter/filter_groups.py SIM04
 ${barnacle_dir}/sample_data/SIM04/Assembly/abyss-1.3.2/barnacle/ver_1.0.0.0/6_with_r2c/S
 IM04.barnacle.data --max-num-groups 3 --no-multi-mapping --no-homopolymers
 --allow-repeats --no-struct-RNA --min-identity 99.0 --min-ctg-rep 0.9 --read-to-contig 5
 --max-ctg-overlap 75 --no-polyA-events
~  ${barnacle_dir}/src/prediction/predict_events.py SIM04
 ${barnacle_dir}/sample_data/SIM04/Assembly/abyss-1.3.2/barnacle/ver_1.0.0.0/7_filtered/S
 IM04.barnacle.pass --transcript-annotations
 ${barnacle_dir}/annotations/ensembl65_ref.txt --transcript-sequences
 ${barnacle_dir}/annotations/Homo_sapiens.GRCh37.65.cdna.all.fa --contig-sequences
 ${barnacle_dir}/sample_data/SIM04/Assembly/abyss-1.3.2/barnacle/ver_1.0.0.0/1_raw_candid
 ates/SIM04.barnacle.contigs
```

${barnacle_dir} represents the directory in which Barnacle is installed.
${cluster_submit_hostname} represents the hostname used to submit cluster jobs.
SIM06 was processed with the same commands as above, replacing “SIM04” with “SIM06”.

S22) TopHat-Fusion commands used for simulation analysis.

```
~  mkdir ${barnacle_dir}/sample_data/tophat_refs
~  cp ${bowtie_install_dir}/scripts/make_hg19.sh ${barnacle_dir}/sample_data/tophat_refs
~  cd ${barnacle_dir}/sample_data/tophat_refs
~  ./make_hg19.sh
~  tophat --bowtie1 --mate-inner-dist -36 --mate-std-dev 12 --num-threads 4
 --fusion-search -o ${barnacle_dir}/sample_data/SIM04/tophat/tophat_out
 ${barnacle_dir}/sample_data/tophat_refs/hg19
 ${barnacle_dir}/sample_data/SIM04/simulated_reads/wildtype_transcripts.r1.fq
 ${barnacle_dir}/sample_data/SIM04/simulated_reads/wildtype_transcripts.r2.fq
~  cd ${barnacle_dir}/sample_data/SIM04/tophat
~  ln -si ${tophat_install_dir}/annotation/*.txt .
~  ln -si ${tophat_install_dir}/annotation/mcl .
~  tophat-fusion-post --num-fus-pairs 0 ${barnacle_dir}/sample_data/tophat_refs/hg19
~  tophat --bowtie1 --mate-inner-dist -36 --mate-std-dev 12 --num-threads 4
 --fusion-search -o ${barnacle_dir}/sample_data/SIM06/tophat/tophat_out
 ${barnacle_dir}/sample_data/tophat_refs/hg19
 ${barnacle_dir}/sample_data/SIM04/simulated_reads/wildtype_transcripts.r1.fq,${barnacle_
 dir}/sample_data/SIM05/simulated_reads/simulated_transcripts.r1.fq
 ${barnacle_dir}/sample_data/SIM04/simulated_reads/wildtype_transcripts.r2.fq,${barnacle_
 dir}/sample_data/SIM05/simulated_reads/simulated_transcripts.r2.fq
~  cd ${barnacle_dir}/sample_data/SIM06/tophat
~  ln -si ${tophat_install_dir}/annotation/*.txt .
~  ln -si ${tophat_install_dir}/annotation/mcl .
~  tophat-fusion-post --num-fus-pairs 0 ${barnacle_dir}/sample_data/tophat_refs/hg19
```

${barnacle_dir} represents the directory in which Barnacle is installed.
${bowtie_install_dir} represents the directory in which Bowtie is installed.
${tophat_install_dir} represents the directory in which TopHat is installed.
